# Myeloid-Derived Suppressor Cells Promote the Progression of Primary Membranous Nephropathy by Enhancing Th17 Response

**DOI:** 10.3389/fimmu.2020.01777

**Published:** 2020-08-20

**Authors:** Huimin Li, Hao Wu, Qiaoyan Guo, Hongyu Yu, Ying Xu, Jinyu Yu, Zhongkun Wang, Huanfa Yi

**Affiliations:** ^1^Central Laboratory, The First Hospital of Jilin University, Changchun, China; ^2^Key Laboratory of Organ Regeneration and Transplantation, Ministry of Education, Changchun, China; ^3^Department of Clinical Laboratory, The Second Hospital of Jilin University, Changchun, China; ^4^Department of Nephrology, The First Hospital of Jilin University, Changchun, China; ^5^Department of Nephrology, The Second Hospital of Jilin University, Changchun, China

**Keywords:** primary membranous nephropathy, myeloid-derived suppressor cells, T helper 2, T helper 17, arginase-1

## Abstract

Several studies have confirmed that the myeloid-derived suppressor cells (MDSCs) are closely associated with autoimmune diseases, but their exact role in these processes remains largely unclear. Here, we investigated the role MDSCs in patients with primary membranous nephropathy (PMN). Compared to healthy controls (HCs), PMN patients showed significantly increased number of HLA-DR^−^CD11b^+^CD33^+^ MDSCs in the peripheral blood, including both CD14^+^CD66b^−^ monocytic and CD14^−^CD66b^+^ granulocytic MDSCs. The frequency of MDSCs was positively correlated with the level of serum anti-phospholipase A2 receptor (anti-PLA2R), 24-h urine protein quantification, and disease activity in PMN patients. Consistently, enhanced T helper 2 (Th2) and T helper 17 (Th17) immune responses were positively associated with plasma anti-PLA2R levels, 24-h urine protein quantification, and the disease activity in PMN patients. Moreover, compared to HCs, MDSCs from PMN patients exhibited significantly elevated arginase-1 (ARG-1) production and increased potential to promote Th17 differentiation *in vitro* in an ARG-1–dependent manner. This study directly demonstrates a pathogenic role for MDSCs in human PMN and provides a molecular mechanism for the pathogenesis of PMN. Our data show that MDSCs may promote PMN disease progression mainly by enhancing Th17 response. Therefore, MDSCs may be an important diagnostic, therapeutic, and prognostic marker for PMN diseases.

## Introduction

Primary membranous nephropathy (PMN), a common cause of the nephrotic syndrome in adults, is an organ-specific autoimmune disease (1–8). Most PMN cases (about 85%) are mediated by antibodies to anti-phospholipase A2 receptor (anti-PLA2R), while the rest are related to thrombospondin type 1 domain containing 7A (anti-THSD7A) or unidentified mechanisms (4, 7, 9, 10). The disease activity of PMN is closely associated with anti-PLA2R level and 24-h urine protein quantification (10–12). PMN occurs in the absence of identifiable causes in most patients. Therefore, a better understanding of the pathogenesis underlying the disease is needed for the identification of new therapeutic targets and strategies.

Some studies have shown that PMN is a Th2-related autoimmune disease (13–15). Compared to controls and other forms of glomerulonephritis, the percentage of IL-4^+^CD4^+^ and IL-10^+^CD4^+^ cells are significantly increased (the former is positively correlated with proteinuria), and the Th1/Th2 ratio is significantly lower in PMN patients (15, 16). Th2 cytokines promote the production of immunoglobulin (Ig) G4 type antibodies by B lymphocytes in PMN (13). In addition, Th17 cells are found in renal biopsies of human glomerulonephritis and renal autoimmunity, and may serve as a therapeutic target in the future (17–19). However, the relationship between Th17 cells and PMN remains unclear.

Myeloid-derived suppressor cells (MDSCs) are heterogeneous immature myeloid cells derived from the bone marrow. They are characterised by a remarkable ability to suppress T-cell responses through the production of reactive oxygen species (ROS), arginase-1 (ARG-1), and nitric oxide (NO) (20). Human MDSCs are CD11b^+^CD33^+^HLA-DR^−^ and can be further classified into two major subsets, the CD14^+^ monocytic MDSCs (M-MDSCs) and the CD15^+^CD66b^+^ granulocytic MDSCs (G-MDSCs) (21). MDSCs have been reported to be involved in autoimmune disease involving Th17 cells including systemic lupus erythematosus (SLE) (22), rheumatoid arthritis (RA) (23, 24), and experimental autoimmune encephalitis (EAE) (25). However, some contradictory observations have been reported concerning the relationship between MDSCs and Th17 cells in some autoimmune diseases, and even in different stages of the same disease. In murine models of collagen-induced arthritis (26) and EAE (27), MDSCs attenuate disease progression by inhibiting the proliferation of CD4^+^ T cells and Th17 response. Conversely, MDSCs promote Th17 polarisation and disease severity due to their pro-inflammatory role in RA (23, 24), SLE (22), and EAE (25). In addition, the relationship between MDSCs and Th2 cells in the context of different diseases is diverse. MDSCs exacerbate Sjögren's syndrome (SS) by inhibiting Th2 cells in human disease and in murine models (28). However, elevated MDSCs are associated with a significant elevation in Th2 cytokine, interleukin-13 (IL-13), and disease prognosis in lung, oesophageal, pancreatic, and gastric cancers (29). Doxorubicin (DOX) treatment promotes breast tumour lung metastasis and tumour angiogenesis by inducing MDSCs to release exosomes miR-126a, to increase Th2 cell response (30).

The role of MDSCs in Th2 and Th17 differentiation and in the pathogenesis of PMN is relatively unknown. Here, our data show that MDSCs are significantly increased in the peripheral blood mononuclear cells (PBMCs) from PMN patients. Enhanced Th2 and Th17 immune responses are positively correlated with the disease activity in PMN patients. Finally, we determined that MDSCs may play an important role in the pathogenesis and progression of PMN by enhancing the Th17 response, which needs to be explored further to develop novel and effective monitoring and therapeutic strategies for PMN.

## Materials and Methods

### Study Design

Twenty-nine PMN patients were randomly recruited to participate in the study between March 2016 and September 2018 from the First and the Second Hospitals of the Jilin University (Tables S1, S2). All patients were diagnosed with PMN both clinically and pathologically by renal biopsy, and none of these patients had received glucocorticoid or immunosuppressant therapy prior to the renal biopsy. We excluded patients with secondary systemic diseases, including tumours, chronic viral, bacterial, and parasitic infections, autoimmune diseases, and those with multiple therapeutic drug use which may cause secondary membranous nephropathy, through clinical and laboratory examinations. The selected patients had not received immunosuppressive therapy within 3 months prior to the start of the study. None of the subjects had any history of immunological diseases or recent infections. Healthy volunteers were randomly recruited as healthy controls (HCs) with an effort to match the age and gender of the PMN patients. We ensured a blinded outcome assessment for all the experiments that used clinical samples. All participants provided written informed consent, and all procedures were approved by the ethics committee of the First Hospital of Jilin University and the ethics committee of the Second Hospital of Jilin University.

### Cell Preparation

PBMCs were prepared by density gradient centrifugation using Lymphoprep (Axis-Shield, Oslo, Norway). Naïve CD4^+^ T cells were isolated from PBMCs using a naïve CD4^+^ T-cell isolation kit II (Miltenyi Biotec, Bergesh-Gladbach, Germany) according to the manufacturer's instructions, and the purity of the cells after separation was >98%.

### Flow Cytometric Analysis

Flow cytometry was used to determine the phenotype of the human MDSCs and T cells using various combinations of the following fluorochrome-conjugated mAbs: anti-human HLA-DR(TU36), CD11b (ICRF44), and CD33 (WM53) were from BD Biosciences; CD14 (M5E2), CD4 (RPA-T4), IFN-γ (4S.B3), CD25 (BC96), and CD66b (G10F5) were from BioLegend; FOXP3 (236A/E7), IL-17A (eBio64DEC17), and IL-4 (8D4-8) were from ThermoFisher Scientific. Isotype controls used included mouse IgG1 (RMG1-1, MOPC-21), IgG2b (MPC-11), and IgM (MM-30) from BioLegend, mouse IgG1 (MOPC-21), IgG2a (R19-15), and IgG2b (27–35) from BD Biosciences. For intracellular staining, the cells were first stained for surface antigens, fixed, permeabilised with intracellular fixation and permeabilisation buffer (eBiosciences, San Diego, CA, USA), followed by staining with fluorochrome-conjugated mAb against the relevant intracellular proteins. All samples were collected on a fluorescence-activated cell sorter (FACS; LSRFortessa, Becton Dickinson, Franklin Lakes, NJ, USA) and analysed using the FlowJo software. Isotype controls and gating strategy for the flow cytometric analyses are presented in the Supplementary Materials (Figures S1, S2).

### Detection of Anti-PLA2R IgG Using ELISA

Human sera were diluted 1:101 in sample buffer and incubated for 30 min. Following three washes with the washing buffer, the bound antibodies were detected by incubation with anti-human-IgG HRP conjugate for 30 min. Then the samples were washed again as described earlier, and tetramethyl benzidine (TMB) substrate was added for 15 min. All incubations were carried out at 25°C. The optical density (OD) was read at 450 nm using an automated spectrophotometer. The standard curve was generated with the absorbance values (linear, y-axis) of five standard sera (2, 20, 100, 500, and 1,500 relative units [RU/mL]) against their corresponding concentrations (logarithm, x-axis). The concentration of the antibody to be tested was calculated based on this standard curve. The samples were set up in duplicates to improve the reliability of the test. The concentration of the antibody was considered positive if the value was ≥20 RU/mL according to the instructions of anti-PLA2R ELISA Kit (IgG; EUROIMMUN).

### MDSC Isolation and T-Cell Suppression

MDSCs were isolated from PBMCs of HCs and PMN patients using a cell sorter (Influx, Becton Dickinson, Franklin Lakes, NJ, USA). CD4^+^ T cells (2 × 10^5^ cells/well) were co-cultured in the absence or presence of autologous MDSCs at 1:1 ratio from HCs or PMN patients with 5 μg/mL plate-bound anti-CD3 mAb (BioXcell, West Lebanon, NH, USA) and 1 μg/mL soluble anti-CD28 mAb (BioLegend, San Diego, California, USA) in 96-well flat-bottom plate for 3 days, and T-cell proliferation was determined by measuring carboxyfluorescein diacetate succinimidyl ester (CFSE).

### Th17 Polarisation

Purified CD4^+^CD45RA^+^ naïve T cells (2–5 × 10^5^ cells/well) were cultured for 6–7 days under Th17 differentiation conditions with plate-bound anti-human CD3, soluble anti-human CD28 mAb, human TGF-β, human IL-6, human IL-23, human IL-1β, anti-IFN-γ, and anti-IL-4. In some experiments, MDSCs (at a 1:1 ratio to the naïve CD4 T cells) and Nω-Hydroxy-nor-L-arginine (nor-NOHA, 300 μM), a specific arginase inhibitor, were added to determine the role of MDSCs and ARG-1 in Th17 cell differentiation. The cells were further stimulated with leukocyte activation cocktail (BD GolgiPlug™) for the final 5 h, and then stained intracellularly with anti-IL-17A. IL-17A concentration in the culture supernatant was measured (in duplicate) using a human IL-17A ELISA kit.

### Th2 Polarisation

Purified CD4^+^ CD45RA^+^ naïve T cells (2 × 10^5^ per well) were cultured for 5 days under Th2 differentiation conditions with plate-bound anti-human CD3 (OKT-3, BioLegend), soluble anti-human CD28 mAb (CD28.2, BioLegend), human rIL-4 (10 ng/ml, PeproTech), and anti-IFN-γ (20 μg/ml, BioLegend). In some experiments, MDSCs (at a 1:1 ratio to the naïve CD4^+^ T cells), nor-NOHA (at 300 μM), and L-NMMA (at 500 μM) were added to determine the role of MDSCs, ARG*-*1, and inducible nitric oxide synthase (iNOS) in Th2 cell differentiation. In addition, MDSCs were cultured alone or with 10 ng/mL rIL-6 or rIL-10 for 24 h, then transferred and co-cultured in Th2 cell differentiation. The cells were further stimulated with leukocyte activation cocktail (BD GolgiPlug™) for 4–6 h, and then stained intracellularly with an anti-IL-4 antibody. Isotype controls and gating strategy for the flow cytometric analyses are presented in the Supplementary Materials (Figure S2).

### Detection of Human Th1/Th2/Th17 Cytokines With BD™ Cytometric Bead Array

IL-2, IL-4, IL-6, IL-10, and IFN-γ protein levels were measured in a single plasma sample from PMN patients using a Human Th1/Th2/Th17 Cytokine Kit. The standards were reconstituted with the assay diluent in a 15-mL conical polypropylene tube. Labelled 12 × 75-mm tubes were arranged in the following order: 1:2, 1:4, 1:8, 1:16, 1:32, 1:64, 1:128, and 1:256 and 300 μL the assay diluent was added to each tube and serial dilution was performed (0, 20, 40, 80, 156, 312.5, 625, 1,250, 2,500, and 5,000 relative units [pg/mL]). The A1–A7 capture beads were pooled and incubated with serum enhancement buffer for 30 min at room temperature. The mixed capture beads, standard dilutions, or plasma samples, and the human Th1/Th2/Th17 PE detection reagent were mixed in equal ratio and incubated for 3 h at room temperature, protected from light. Wash buffer was added to each assay tube and centrifuged. The supernatant was carefully aspirated and discarded, and the beads pellet was resuspended in 300 μL of wash buffer, and then analysed by flow cytometry. All data were processed using the FCAP Array software.

### Multiplex Immunohistochemistry (IHC) and Immunofluorescence Staining

Renal tissues from PMN patients were fixed in 10% buffered formalin. Paraffin sections were prepared and stained with hematoxylin-eosin (H&E), periodic acid-Schiff (PAS), periodic acid-silver metheramine (PASM), and Masson. For immunofluorescence analysis, tissue sections were prepared and stained with rabbit anti-IL-17 (Abcam, Cambridge, UK) or biotin anti-mouse/human CD11b (BioLegend, San Diego, California, USA) antibodies, or corresponding control antibodies (rabbit IgG and Rat IgG2b for IL-17 and CD11b, respectively), followed by staining with Alexa Fluor 488-conjugated anti-rabbit IgG and streptavidin-Cy3. Human IgG in the frozen tissue sections was determined by staining with Alexa Fluor 488-conjugated anti-human IgG (Dako, Glostrup, Denmark). Multiplexed IHC staining was performed on paraffin-embedded renal sections to determine the association between MDSCs and immune cells using Opal™ Multi-colour IHC kit (PerkinElmer, Waltham, MA, USA) according to the manufacturer's instruction. The isotype control antibodies used for immunofluorescent staining and negative control used for multiplex IHC are presented in the Supplementary Materials (Figure S3).

Briefly, the tissue sections were dewaxed and rehydrated. Antigen retrieval and quenching of endogenous peroxidase activity were performed with AR6 buffer using microwave treatment. The slides were washed and blocked with PerkinElmer Antibody Diluent/Block buffer, followed by primary antibody staining. Then, the slides were washed and incubated in the Opal polymer HRP Ms + Rb for 10 min and visualised using tyramide signal amplification (TSA) dye. Following that, the slides were microwave-treated in AR6 buffer to remove the antibodies and introduce the next primary antibody. Once the antibody staining was complete, the slides were stained with 4,6-diamidino-2-phenylindole (DAPI) and cover-slips were applied with Prolong Gold Antifade Reagent. In addition, single-colour control staining with each primary antibody was also prepared. The primary antibodies are detailed in Table S3. The slides were imaged and processed using Zeiss LSM880 and Olympus FV1000 confocal microscopes.

### Quantification of Plasma ARG-1 and IL-13

The level of plasma ARG-1 and IL-13 were measured using an ELISA kit according to the manufacturer's instructions (Raybiotech, Guang Zhou, China).

### Quantitative Real-Time PCR

RNA was extracted using AxyPrep™ Multisource Total RNA Miniprep Kit, and cDNA was synthesised using SuperScript II Reverse Transcriptase. All PCRs were performed in triplicate and carried out on an ABI StepOnePlus system with TransScript Green Two-Step qRT-PCR SuperMix. The mRNA expression levels were quantified using primers for *ARG-1*, and *GATA3*, and actin was used as an internal control for normalization using the standard 2^−ΔΔCT^ calculation as described previously (31). The primer sequences are as follows: human *ARG-1*, 5′-GTTTCTCAAGCAGACCAGCC-3′(forward) and 5′- GCTCAAGTGCAGCAAAGAGA-3′(reverse); human *GATA3*, 5′-ACCACAACCACACTCTGGAGGA-3′(forward) and 5′-TCGGTTTCTGGTCTGGATGCCT-3′(reverse); and human actin, 5′-TTCAACACCCCAGCCATG-3′ (forward) and 5′-CCTCGTAGATGGGCACAGT-3′ (reverse).

### Statistical Analysis

Statistical analyses were performed using the GraphPad Prism 6.0 software. Data are expressed as means ± standard deviation (*SD*) or median (25%, 75%). Between-group comparisons were performed using a two-tailed *t*-test or a Mann-Whitney *U*-test. Multi-group comparative study was analysed using one-way ANOVA. The Pearson rank test was used for correlation analysis of normally distributed data, and the Spearman rank test was used for the analysis of correlation between non-normally distribution data. *P* < 0.05 was considered significant.

## Results

### A Positive Correlation Between the Number of Circulating MDSCs and Disease Activity in PMN Patients

**Figure 1:**
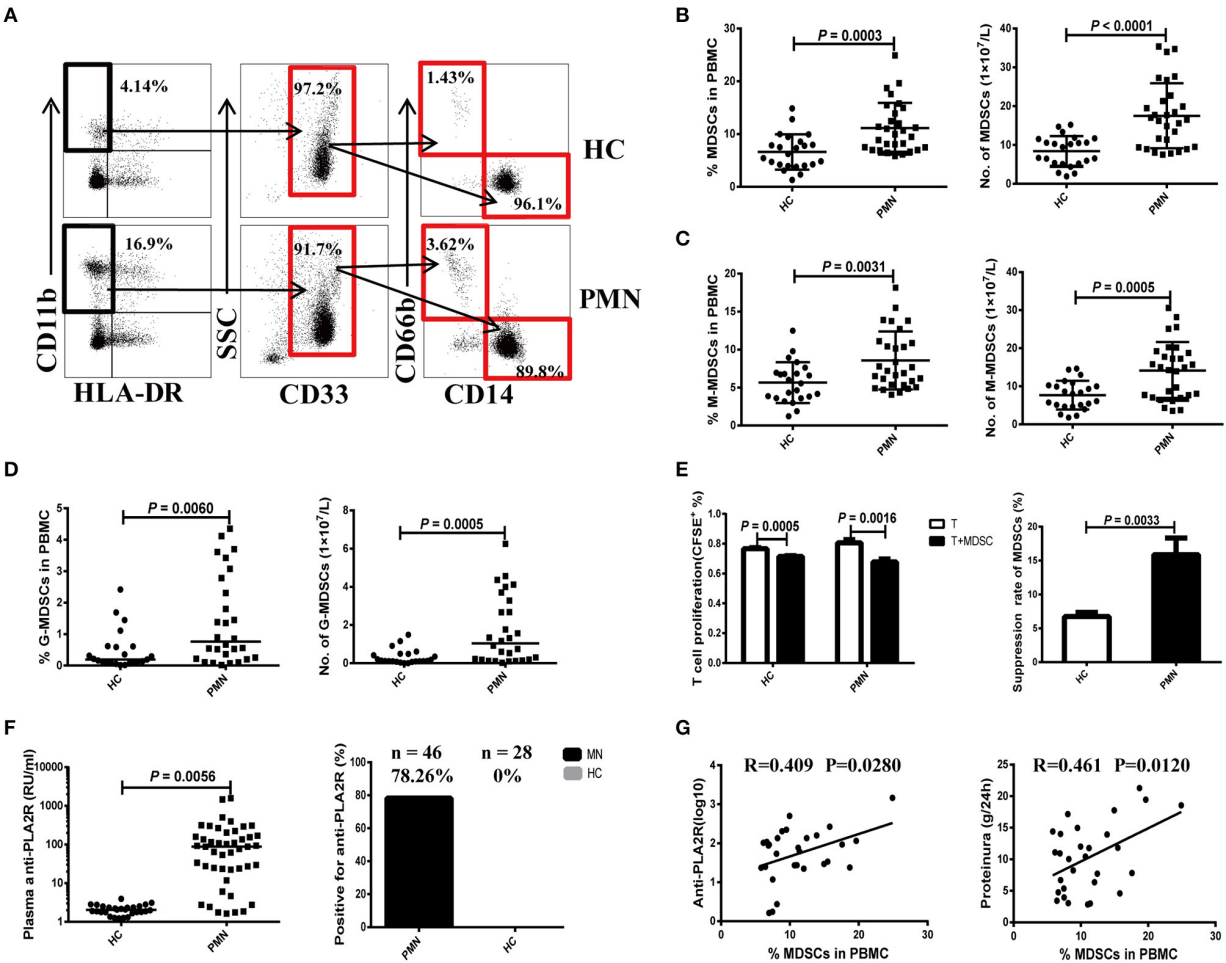
Expansion of MDSCs in PBMCs is correlated with the disease activity in PMN patients. **(A)** Staining profiles of MDSCs (CD11b^+^CD33^+^HLA-DR^−^), M-MDSCs (CD11b^+^CD33^+^HLA-DR^−^CD14^+^CD66b^−^), and G-MDSCs (CD11b^+^CD33^+^HLA-DR^−^CD14^−^CD66b^+^) from representative HC and PMN patients. **(B–D)** Percentage (left panel) and number (right panel) of MDSCs (*P* = 0.0003, *t-*test; *P* < 0.0001, *t-*test, respectively) **(B)**, M-MDSCs (*P* = 0.0031, *t-*test; *P* = 0.0005, *t-*test, respectively) **(C)**, and G-MDSCs (*P* = 0.0060, Mann-Whitney *U*-test; *P* = 0.0005, Mann-Whitney *U*-test, respectively) **(D)** in HCs (*n* = 23) and PMN patients (*n* = 29). **(E)** *In vitro* suppression assay (left panel) revealed that MDSCs from PMN patients have a stronger ability to suppress autologous naïve CD4^+^T cells (anti-CD3/CD28–induced polyclonal T cells) proliferation than those from HCs. T-cell proliferation inhibition rates (right panel) of MDSCs from HC and PMN patients were 6.7 and 15.9%, respectively (*P* = 0.0033, *t-*test). **(F)** Content (*P* = 0.0056, *t-*test; left panel) and positive percentage (right panel) of plasma anti-PLA2R antibody from PMN patients (78.26%, *n* = 46) and HCs (0%, *n* = 28). **(G)** Correlation analysis between MDSC frequency and anti-PLA2R level (*P* = 0.028, Pearson correlation; left panel) or 24-h urine protein quantification (*P* = 0.012, Pearson correlation; right panel) from PMN patients. Anti-PLA2R is displayed with log_10_. The data are shown as mean ± standard deviation (*SD*) or median (25%, 75%). Between-group comparisons were performed using a two-tailed *t*-test for normally distributed data, or a Mann-Whitney *U*-test for non-normally distribution data.

We first measured the frequency of MDSCs and their subsets isolated from PBMCs of PMN patients using flow cytometry. PBMCs were collected from a total of 29 patients (20 males and nine females, aged 22–70 years) and 23 HCs (17 males and six females, aged 25–58 years). Detailed clinical and laboratory characteristics of these patients are presented in Tables S1, S2. MDSCs were defined as CD11b^+^*CD*33^+^*HLA*−*DR*^−^ and were further divided into the SSC^l^°^w^*CD*14^+^*CD*66*b*^−^ (M-MDSC) and SSC^high^*CD*14^−^*CD*66*b*^+^ (G-MDSC) subsets (Figure 1A). Compared to HCs, PMN patients showed significant increase in both the percentage and number of MDSCs (Figure 1B). The percentages and numbers of M-MDSCs (Figure 1C) and G-MDSCs (Figure 1D) in PMN patients were also higher compared to that in HCs. MDSCs isolated from the PBMCs of PMN patients had a stronger ability to suppress the proliferation of autologous naïve CD4^+^T cells than those from the PBMCs of HCs (Figure 1E). Beck et al. (1) confirmed that PLA2R is a major target antigen in PMN patients. Thus, we measured the anti-PLA2R IgG in the plasma in PMN patients and HCs. The plasma content of anti-PLA2R was higher in the PMN patients than in HCs, and a positive percent of anti-PLA2R IgG from PMN was also near 78.26%, but healthy volunteers were negative for anti-PLA2R IgG (<20 RU/mL; Figure 1F). In addition, both the percentage and number of MDSCs were positively correlated with anti-PLA2R and 24-h urine protein quantification (Figure 1G and Figure S4).

### Increased Plasma ARG-1 Content and ARG-1 Production by MDSCs in PMN Patients

**Figure 2:**
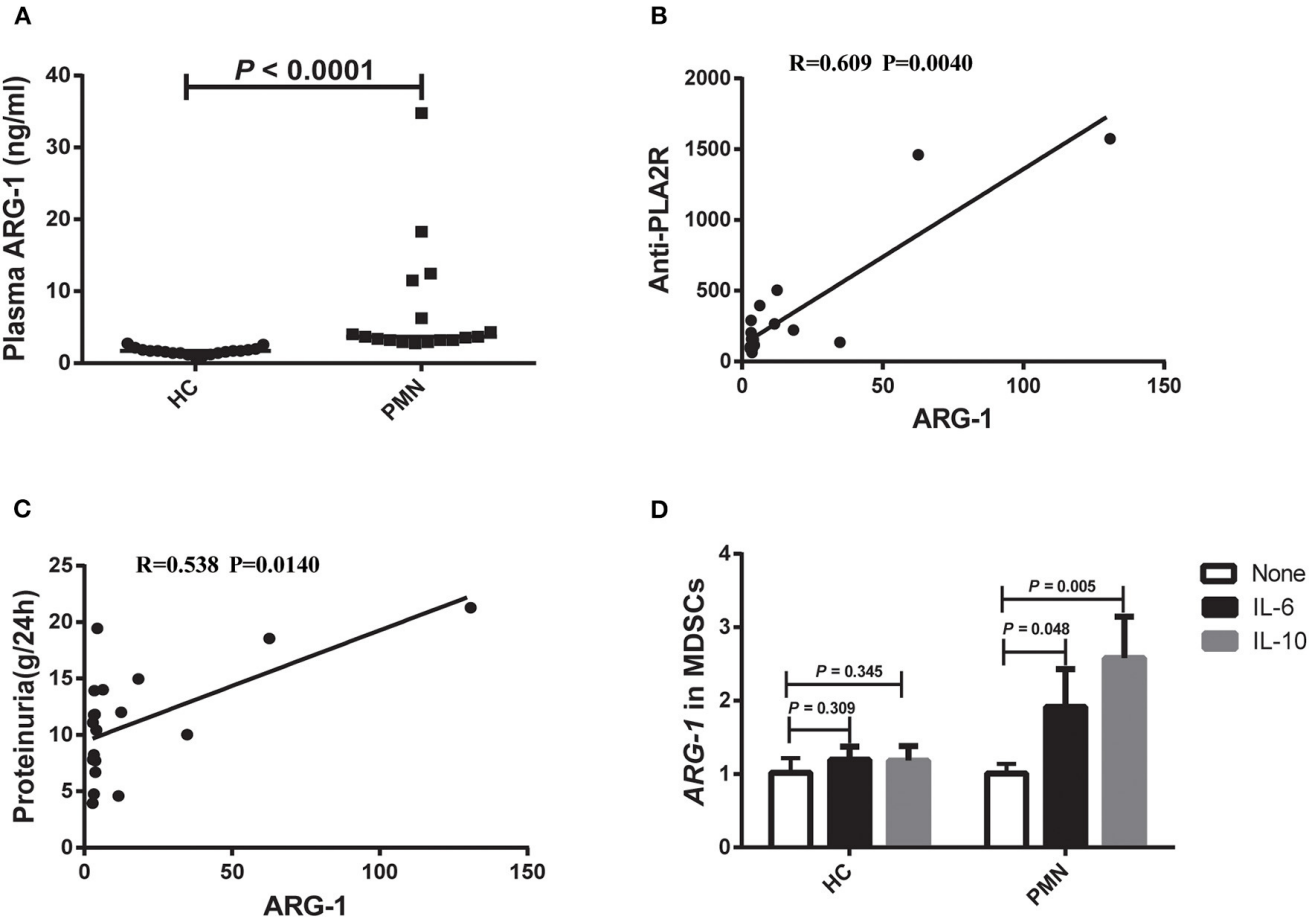
Plasma ARG-1 content is correlated with disease activity in PMN patients. **(A)** Plasma arginase-1 content in HCs and PMN patients (*P* < 0.0001, Mann-Whitney *U*-test; 1.708 [(1.429, 1.960) vs. 3.696 (3.216, 12.204)]. The data are shown as median (25%, 75%). **(B,C)** Positive correlation between plasma ARG-1 from PMN patients and anti-PLA2R levels (*P* = 0.0040, Spearman correlation) **(B)** or 24-h proteinuria quantification (*P* = 0.0140, Spearman correlation) **(C)**. **(D)** MDSCs from HCs and PMN patients were cultured separately for 12–24 h with GM-CSF, then human IL-6 or IL-10 was added, and the level of *ARG-1* mRNA was measured in triplicate using qRT-PCR in HCs (none vs. IL-6, *P* = 0.309; none vs. IL-10, *P* = 0.345, ANOVA) and PMN patients (none vs. IL-6, *P* = 0.048; none vs. IL-10, *P* = 0.005, ANOVA).

ARG-1 is one of the main factors in MDSC-mediated immune suppression (32). Therefore, we measured the plasma ARG-1 content in HCs and PMN patients. The results showed that there was a significant increase in plasma ARG-1 content in PMN patients compared to HCs (Figure 2A). There was a positive correlation between the plasma ARG-1 content and both the anti-PLA2R level (Figure 2B) and the 24-h urine protein quantification (Figure 2C). IL-6 promotes ARG-1 production in MDSCs from SLE patients (22) and induces ARG-1 production in mouse macrophages (33). To determine whether IL-6 and IL-10 are involved in the observed upregulation of ARG-1 in MDSCs from PMN patients, MDSCs from HCs and PMN patients were cultured separately for 12–24 h with GM-CSF, and human IL-6 or IL-10 was added. Real-time quantitative polymerase chain reaction (qPCR) revealed a significant increase in *ARG-1* mRNA expression in MDSCs from PMN patients following treatment with IL-6 and IL-10; however, the difference between the *ARG-1* mRNA expression in MDSCs from HCs was not significant (Figure 2D).

### Increased Number of Th17 Cells and Plasma Level of Th17 Cytokines in PMN Patients

**Figure 3:**
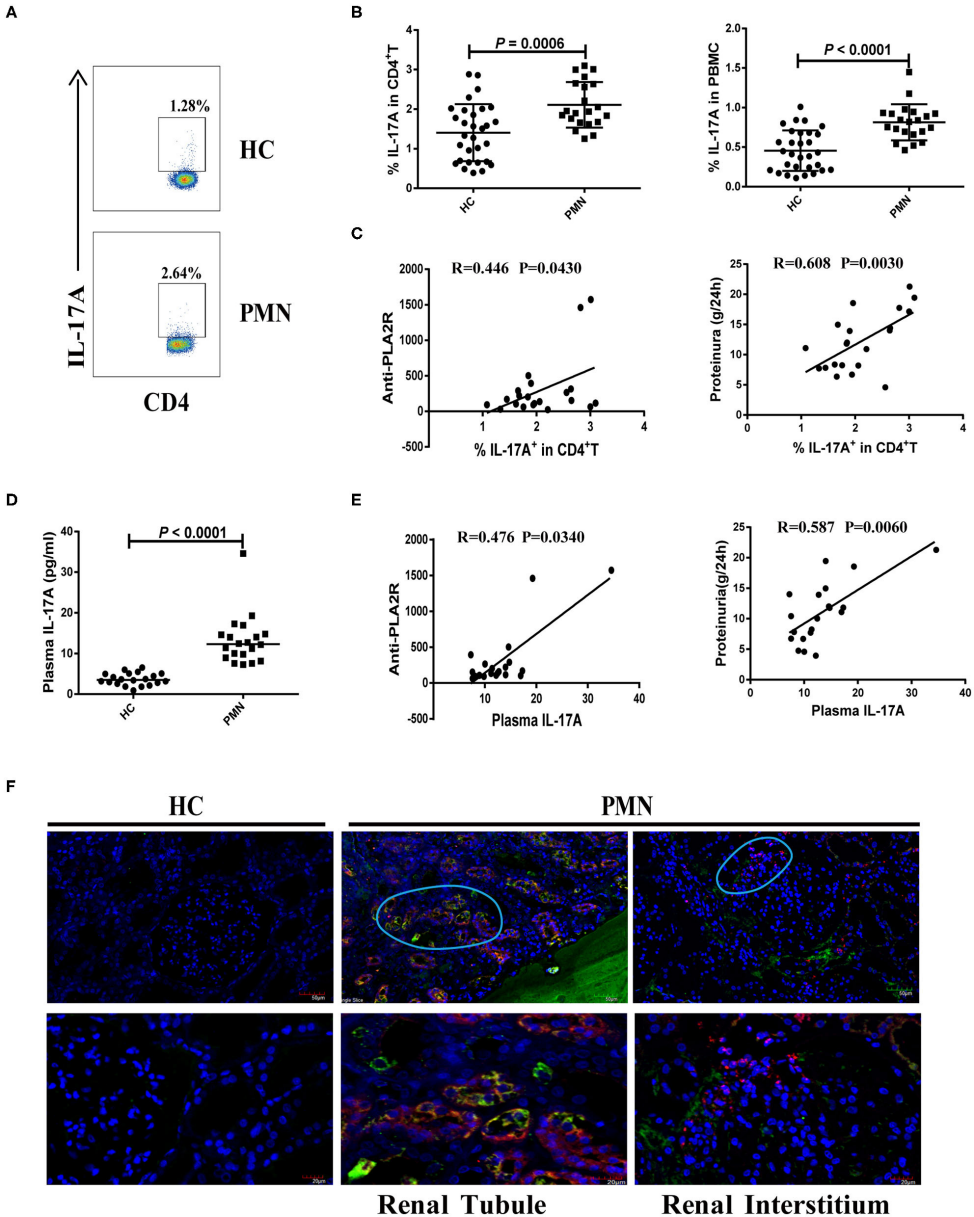
Increased number of Th17 cells and plasma level of Th17 cytokines in PMN patients. Frequency of IL-17^+^CD4^+^ cells in HCs and PMN patients determined using flow cytometry following a brief stimulation with leukocyte activation cocktail. **(A)** Staining profile of IL-17A from the peripheral blood of HCs and PMN patients. **(B)** Percentage of IL-17A (1.406 ± 0.722% vs. 2.107 ± 0.577%; 0.455 ± 0.256% vs. 0.814 ± 0.228%) in CD4^+^T (left panel) and PBMCs (right panel) from peripheral blood of HCs and PMN patients. **(C)** Correlation analysis between IL-17^+^CD4^+^ T cell frequency and anti-PLA2R levels (*P* = 0.0430, Spearman correlation; left panel) or 24-h proteinuria quantification (*P* = 0.0030, Spearman correlation; right panel). **(D)** Plasma IL-17A content from HCs and PMN patients (*P* < 0.0001, Mann-Whitney *U*-test; 3.525 [(2.640, 5.045) vs. 12.317 (9.159, 14.735)]. Data are shown as mean ± *SD* or median (25%, 75%). **(E)** Positive correlation between plasma IL-17A and anti-PLA2R levels from PMN patients (*P* = 0.0340, Spearman correlation; left panel) or 24-h proteinuria quantification (*P* = 0.0060, Spearman correlation; right panel). **(F)** Immunofluorescent staining of IL-17 and CD11b in kidney tissue sections from two patients with minimal change disease and a healthy subject used as a control (*n* = 3) and PMN patients (*n* = 3) using anti-human CD11b (red)/IL-17 (green) antibody and DAPI (blue). IL-17^+^ cells were adjacent to CD11b^+^ cells and were primarily detected in the renal tubules (PMN-left panel) and interstitial areas (PMN-right panel) of PMN patients. Representative images are shown. Scale bars represent 50 μm (top) and 20 μm (bottom), respectively.

We analysed the frequency of Th17 cells (CD4^+^ T cells that produce IL-17A) following a brief stimulation with leukocyte activation cocktail (BD GolgiPlug™) using a flow cytometry-based intracellular cytokine detection method and plasma IL-17A content using by ELISA. We also analysed their association with anti-PLA2R level and the degree of proteinuria in PMN patients. Patients with PMN showed significantly increased frequency of IL-17A^+^ CD4^+^T cells (Figure 3A) compared to HCs (Figures 3A,B), which was positively correlated with anti-PLA2R level (*P* = 0.0430, Spearman correlation) and the severity of 24-h proteinuria quantification (*P* = 0.0030; Spearman correlation; Figure 3C). In agreement with that, the IL-17A plasma content in PMN patients increased significantly compared with HCs (Figure 3D), and there was a positive correlation between the anti-PLA2R level and 24-h proteinuria quantification (Figure 3E). Immunofluorescent staining revealed the presence of IL-17^+^ and CD11b^+^ cells in the renal biopsy samples from all PMN patients (*n* = 3; Figure 3F). IL-17^+^ cells were adjacent to the CD11b^+^ cells and were primarily detected in the renal tubules and interstitial areas (Figure 3F). Collectively, our data suggest that Th17 cells and cytokines are likely to play an important role in the pathogenesis of PMN.

### Increased Potential of MDSCs From PMN Patients to Promote Th17 Cell Differentiation *in vitro* Through an ARG-1-Dependent Mechanism

**Figure 4:**
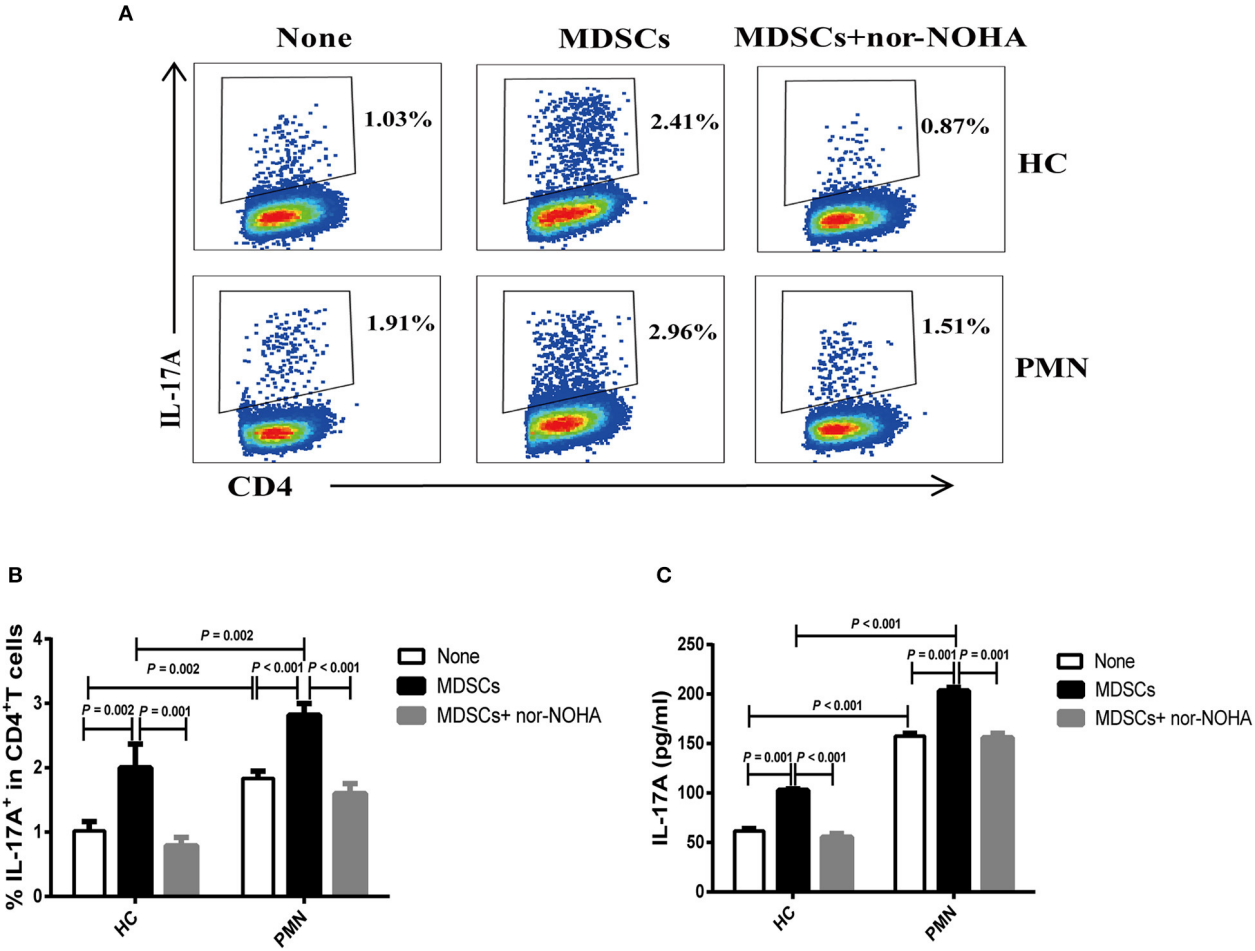
MDSCs facilitate Th17 cell differentiation *in vitro* in an ARG-1-dependent manner. Naïve CD4^+^T cells from HCs and PMN patients were cultured under Th17 differentiation condition without MDSCs, with autologous MDSCs, or with autologous MDSCs + nor-NOHA and analysed for the expression of Th17 and relevant cytokines. Shown are representative staining profiles **(A)** and percentage **(B)** of IL-17A^+^ CD4^+^ T cells from HCs (*n* = 3; Th17 vs. MDSC, *P* = 0.002; MDSC vs. MDSC + nor-NOHA, *P* = 0.001, ANOVA) and from PMN patients (*n* = 3; Th17 vs. MDSC, *P* < 0.001; MDSC vs. MDSC + nor-NOHA, *P* < 0.001, ANOVA) and (HC-None vs. PMN-None, *P* = 0.002; HC-MDSCs vs. PMN-MDSCs, *P* = 0.002, ANOVA). **(C)** Levels of IL-17A from HCs (*n* = 3; Th17 vs. MDSC, *P* = 0.001; MDSC vs. MDSC + nor-NOHA, *P* < 0.001, ANOVA) and from PMN patients (*n* = 3; Th17 vs. MDSC, *P* = 0.001; MDSC vs. MDSC + nor-NOHA, *P* = 0.001, ANOVA) and (HC-None vs. PMN-None, *P* < 0.001; HC-MDSCs vs. PMN-MDSCs, *P* < 0.001, ANOVA) in the culture supernatants measured by ELISA. Data shown are representative of three independent experiments.

MDSC-derived ARG-1 promotes Th17 differentiation and is associated with the disease activity in SLE (22). We assessed the effect of MDSCs in Th17 cell polarising medium consisting of IL-6, TGF-β, IL-23, IL-1β, anti-IFN-γ, and anti-IL-4 monoclonal antibodies (mAbs) (22, 34). The results showed that MDSCs significantly promoted the generation of CD4^+^ T cells that secrete IL-17A. MDSCs from PMN patients were significantly more potent than those from HCs in promoting Th17 cell differentiation, and MDSC-mediated Th17 differentiation was completely abrogated in the presence of nor-NOHA, suggesting that the effect of MDSCs was ARG-1-dependent (Figures 4A–C).

### Enhanced Th2 Response Is Positively Correlated With the Disease Activity of PMN Patients

**Figure 5:**
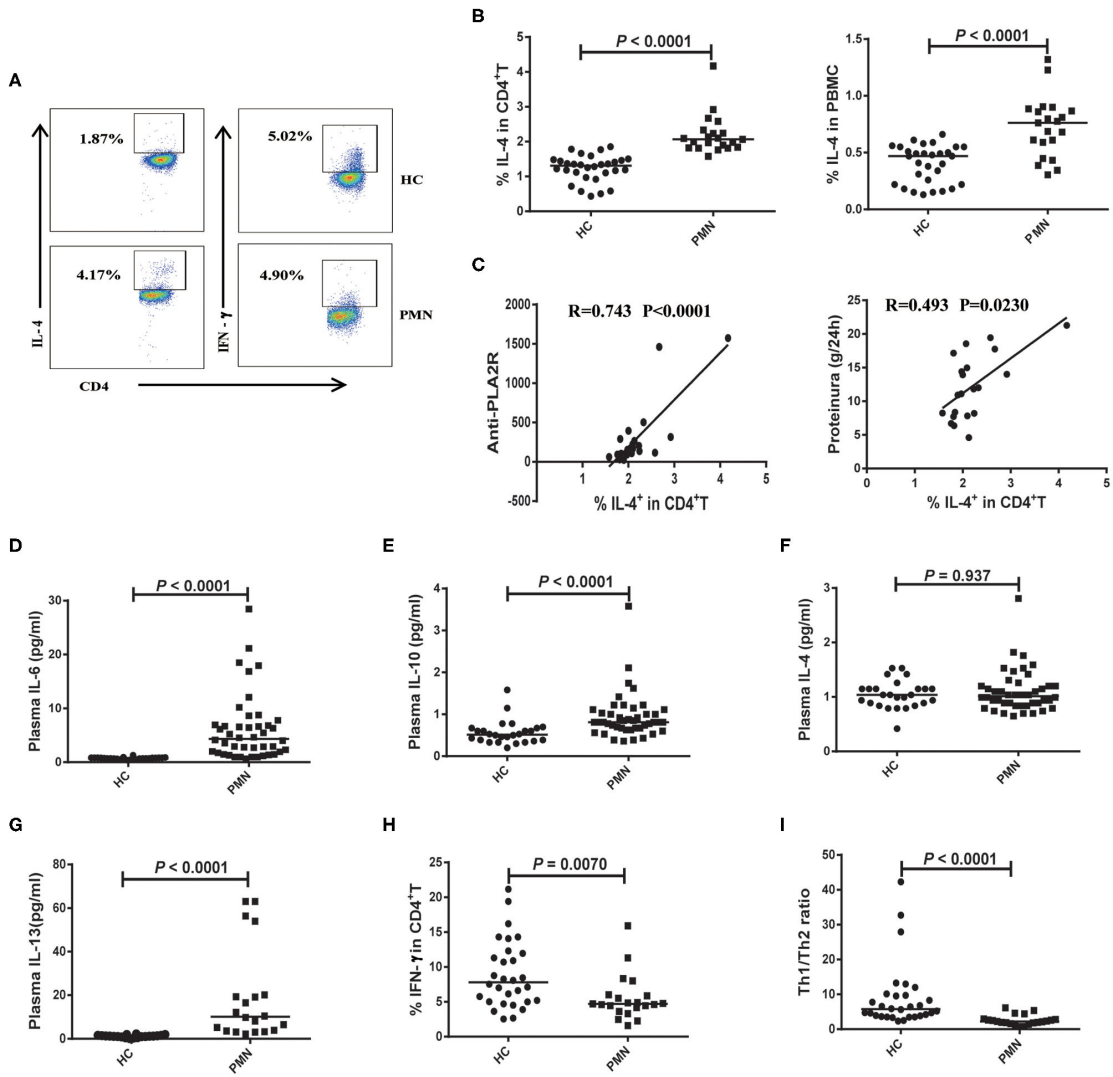
Enhanced Th2 response is probably associated with the disease activity of PMN patients. PBMCs were prepared from PMN patients and HCs, and frequencies of IL-4^+^CD4^+^ T and IFN-γ+ CD4^+^ T cells were determined using flow cytometry following a brief stimulation with leukocyte activation cocktail. **(A)** Staining profile of IL-4 and IFN-γ from the peripheral blood of HCs and PMN patients. **(B)** Percentage of IL-4 [1.230 (1.040, 1.530) vs. 2.130 (1.903, 2.455); 0.449 (0.270, 0.527) vs. 0.859 (0.769, 0.902)] in CD4^+^T (left panel) and PBMCs (right panel) from peripheral blood of HCs and PMN patients. **(C)** Correlation analysis between IL-4^+^CD4^+^ cell frequency and anti-PLA2R levels (*P* < 0.0001, Spearman correlation; left panel) or 24-h proteinuria quantification (*P* = 0.0230, Spearman correlation; right panel). **(D)** IL-6 (*P* < 0.0001, Mann-Whitney *U*-test; [0.720 (0.580, 0.840) vs. 4.020 (1.595, 7.860)], **(E)** IL-10 (*P* < 0.0001, Mann-Whitney *U*-test; [0.530 (0.390, 0.670) vs. 0.850 (0.775 1.070)], **(F)** IL-4 (*P* = 0.937, Mann-Whitney *U*-test; [1.040 (0.865, 1.150) vs. 1.040 (0.890, 1.335)] and **(G)** IL-13 (*P* < 0.0001, Mann-Whitney *U*-test; [1.131 (0.728, 1.472) vs. 8.996 (3.484, 28.638)] content in plasma from HCs and PMN patients, respectively, measured using BD CBA and ELISA kits. **(H)** Percentage of IFN-γ [7.010 (4.138, 19.170) vs. 4.695 (1.854, 14.060)] in CD4^+^T from peripheral blood of HCs and PMN patients. **(I)** Th1/Th2 ratio (*P* < 0.0001, Mann-Whitney *U*-test; [6.456 (3.527, 9.932) vs. 2.248 (1.712, 2.896)] in the HCs and PMN. The data are shown as median (25%, 75%). Each symbol represents an individual HC or patient. The statistical significance of differences between groups was determined using the Pearson correlation (bivariate normal distribution) or Spearman's rank correlation (bivariate non-normal distribution; *P* < 0.05).

The polarisation of the T cell subsets, Th1 and Th2, has been established to be important in animal models and human autoimmune diseases (35). Th2 responses are associated with membranous injury in renal disease (36). Th1 cells produce IL-2 and IFN-γ cytokines. Th2 cells produce IL-4, IL-6, IL-10, and IL-13 cytokines (37, 38). PBMCs were prepared from PMN patients and HCs, and the frequencies of Th2 (CD4^+^ T cells that produce IL-4) and Th1 (CD4^+^ T cells that produce IFN-γ) cells were determined using flow cytometry following a brief activation. Compared to HCs, the frequency of IL-4^+^CD4^+^ cells were significantly increased (Figures 5A,B) in PMN patients, and positively correlated with anti-PLA2R levels (*P* < 0.0001, Spearman correlation) and the severity of 24-h proteinuria quantification (*P* = 0.0230; Spearman correlation) (Figure 5C). In agreement with that, plasma levels of IL-6, IL-10, and IL-13 in PMN patients were found to be significantly higher compared to those in HCs (Figures 5D,E,G), although IL-4 level was not significantly different in PMN patients and HCs (Figure 5F). Conversely, we observed decreased frequency of IFN-γ^+^CD4^+^ cells in the PMN patients compared to HCs (Figure 5H), while there was no significant difference in IFN-γ and IL-2 between PMN patients and HCs (data not shown). The Th1/Th2 ratio was significantly lower in PMN compared to the HCs (Figure 5I). Since Zhu et al. reported that regulatory T cells (Tregs) suppress Th1 and Th2 responses (39), we analysed the expression of Tregs in the PBMCs from PMN patients and HCs. The results showed that the Treg percentages in the CD4^+^T cells from HCs were higher than that from PMN patients. However, the number of Tregs were not different between HCs and PMN patients (Figure S5).

### CD3^+^ T Cells Were Adjacent to CD11b^+^ Cells in PMN Patients

**Figure 6:**
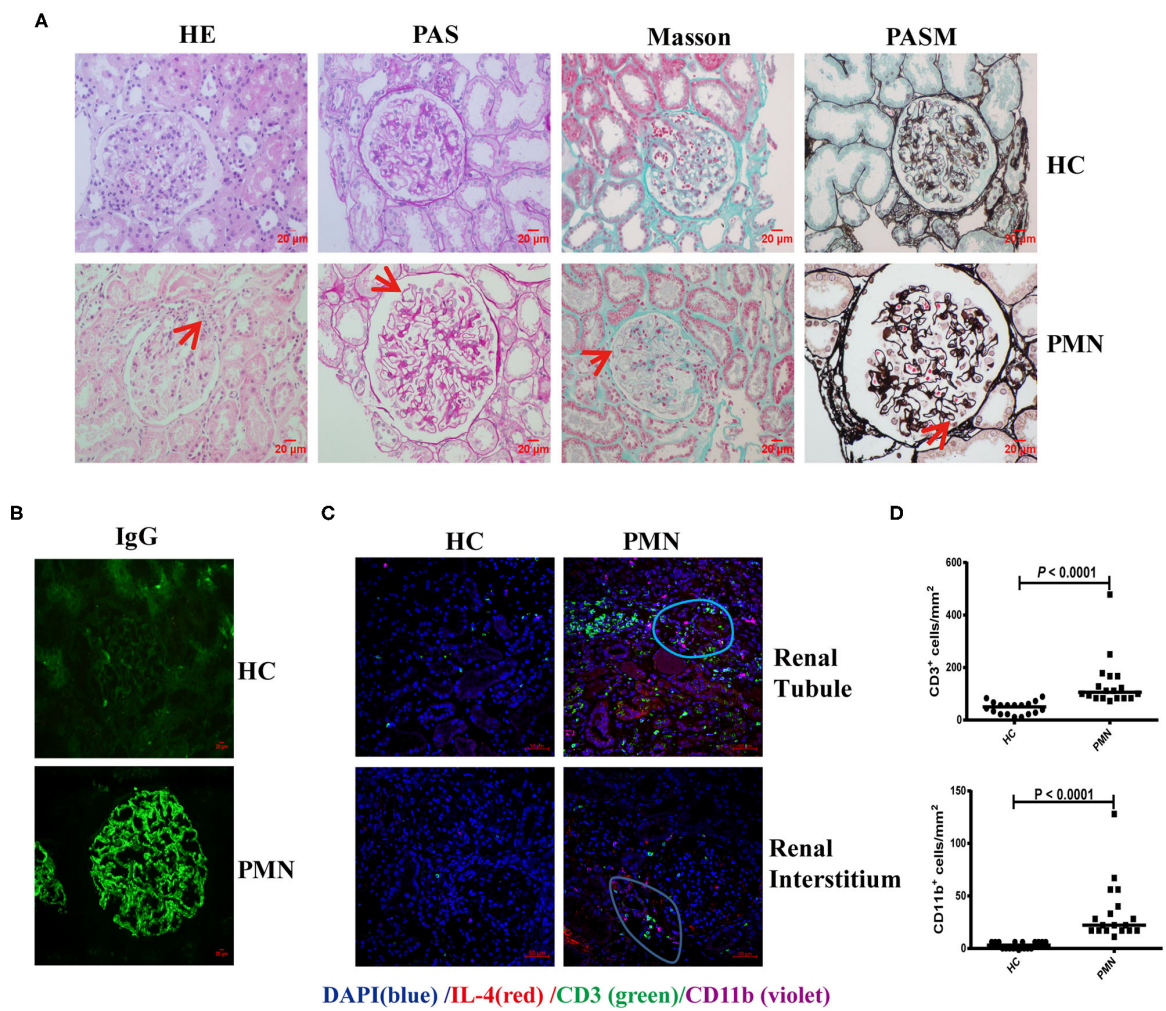
CD3^+^ T cells were adjacent to CD11b^+^ cells in PMN patients. Histological analysis of renal biopsy samples from PMN patients (*n* = 3). Renal biopsy samples from two patients with minimal change disease and a healthy subject were used as controls (*n* = 3). **(A)** Pathological features of the kidney tissues from PMN patients shown using light microscopy (LM). Focal inflammatory cell infiltration outside the arteriolar capsule (arrow) in the H&E-stained section, mesangial proliferation in the glomerulus (arrow) identified by PAS staining, immune complex deposition and “spikes” displayed in the basal membrane by Masson and PASM are shown. Representative images are shown. Original magnification, ×400. **(B)** Immunofluorescence (IF) staining of IgG in kidney tissue sections from HCs and PMN patients. Original magnification, ×400. **(C)** The kidney tissues from PMN patients were analysed by multiplex IHC assays using anti-human IL-4 (red)/CD3 (green)/CD11b (violet) antibody and DAPI (blue). The result shows that IL-4 is diffusely expressed in the kidney tissue. Representative staining images of kidney tissues from PMN patients are shown. Scale bars represent 50 μm. **(D)** The densities (per mm^2^) of CD3^+^ and CD11b^+^ cells in HCs and PMN patients, and the randomly selected areas of the kidney tissues from HCs and PMN patients. Each symbol represents the density of positively stained cells in each individual microscopic area (six areas were counted for each kidney sample, and data were analysed using the Mann-Whitney *U*-test).

Membranous nephropathy is characterised by the deposition of a large amount of immune complex on the epithelial side of the glomerular capillary loop. H&E staining revealed a small amount of inflammatory cell infiltration in the interstitium and outside the capsule arteriolar capsule. PAS staining showed stiffening and thickening of glomerular capillary loops, as well as tubular atrophy. Diffuse immune complex deposition on the basement membrane was observed by Masson staining; PASM staining revealed “nail spikes,” which result from the matrix composition extending out of a damaged basement membrane (Figure 6A). IgG was deposited as coarse particles along the glomerular capillary wall with typical immunofluorescence (Figure 6B). Kidney tissues from PMN patients were analysed using *in situ* quantitative multiplex IHC assays using anti-human IL-4, CD3, and CD11b antibodies (Figure 6C). The result showed that IL-4 was diffusely expressed in the kidney tissue, and further confirmed the presence of CD3^+^ T cells adjacent to CD11b^+^ cells in the renal tubule and interstitial areas in the PMN patients. The densities of CD3^+^ and CD11b^+^ cells in the IL-4- enriched or T cells-enriched areas in PMN patients were significantly higher than in the same areas in HCs (Figure 6D). Collectively, these data suggest that the interaction of CD3^+^ T cells and CD11b^+^ cells plays an important role in the onset and development of PMN.

### IL-6 and IL-10 Enhance the Ability of MDSCs to Inhibit Th2 Differentiation *in vitro*

**Figure 7:**
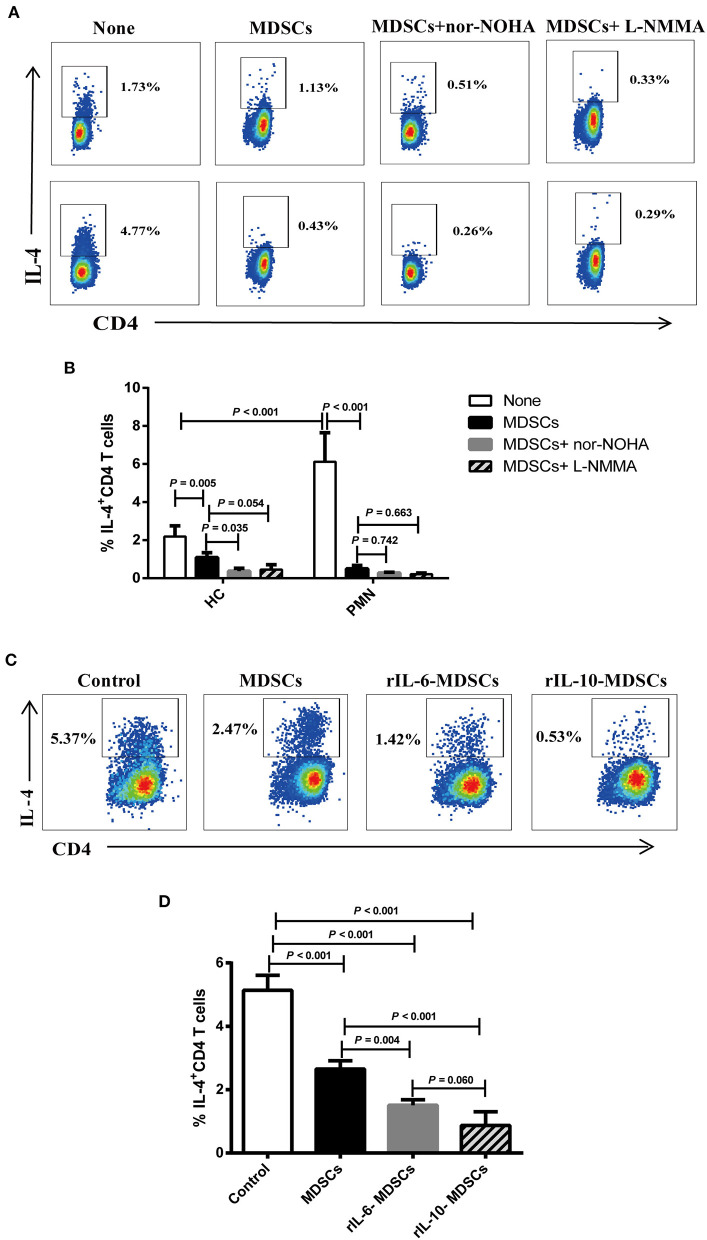
IL-6 and IL-10 enhance the ability of MDSCs to inhibit Th2 differentiation *in vitro*. Naïve CD4^+^ T cells from HCs and PMN patients were cultured under Th2 differentiation condition without MDSCs, with autologous MDSCs, with autologous MDSCs + nor-NOHA or with autologous MDSCs + L-NMMA, or with MDSCs alone, MDSCs with 10 ng/mL rIL-6, or MDSCs with 10 ng/mL rIL-10, and analysed the expression of IL-4^+^CD4^+^ T cell. **(A)** Representative staining profiles, and **(B)** percentages of IL-4^+^CD4^+^ T cells from HCs (Th2 vs. MDSCs, *P* = 0.005; MDSCs vs. MDSCs + nor-NOHA, *P* = 0.035; MDSCs vs. MDSCs + L-NMMA, *P* = 0.054, ANOVA) and PMN patients (*n* = 3; Th2 vs. MDSCs, *P* < 0.001; MDSCs vs. MDSCs + nor-NOHA, *P* = 0.742; MDSCs vs. MDSCs + L-NMMA, *P* = 0.663, ANOVA), and HC-Th2 vs. PMN-Th2, *P* < 0.001, ANOVA). **(C)** Representative staining profiles, and **(D)** percentages of IL-4^+^ CD4^+^ T cells (*n* = 3; Th2 vs. MDSCs, *P* < 0.001; Th2 vs. 10 ng/mL rIL-6 MDSCs, *P* < 0.001; Th2 vs. 10 ng/mL rIL-10 MDSCs, *P* < 0.001; MDSCs vs. 10 ng/mL rIL-6 MDSCs, *P* = 0.004; MDSCs vs. 10 ng/mL rIL-10 MDSCs, *P* < 0.001; 10 ng/mL rIL-6 MDSCs vs. 10 ng/mL rIL-10 MDSCs, *P* = 0.060, ANOVA) by intracellular staining of Th2 cells from PMN patients. Data shown are representative of three independent experiments. Data are expressed as the median and range from three independent experiments.

ARG-1 and iNOS, which are regulated by Th1 and Th2 cytokines, respectively, are the main factors in MDSC-mediated immune suppression (40). We assessed the effect of MDSCs on Th2 cell differentiation from anti-CD3/CD28-activated naïve CD4^+^ T cells in Th2 polarising medium consisting of rIL-4 and anti-IFN-γ antibody (41–43). To further verify that the IL-4^+^ cells were indeed Th2 polarised cells, we analysed the expression of endothelial transcription factor 3, GATA binding protein 3 (GATA3), a specific transcription factor of the Th2 cells under Th2 differentiation without MDSCs or with autologous MDSCs using qRT-PCR. The results showed that the mRNA expression of *GATA3* in Th2 cells with autologous MDSCs were lower than that without MDSCs from HCs and PMN patients, and *GATA3* mRNA expression in Th2 cells with autologous MDSCs from PMN patients were significantly lower than that from HCs (Figure S6A). We found that PMN patients had significantly increased frequency of IL-4^+^CD4^+^ cells compared to HCs, indicating that T cells from PMN patients tended to be more easily polarised into Th2 cells, and MDSCs from both HCs and PMN patients inhibited naïve CD4^+^ T differentiation into Th2 cells (Figures 7A,B). However, the inhibitory effect of MDSC-mediated Th2 differentiation was not restored in by nor-NOHA, a selective ARG-1 inhibitor, or L-NMMA, an iNOS inhibitor (Figure 7B), or both (Figures S6B,C), suggesting that the effects of MDSCs were not mediated by ARG-1 or iNOS production.

Cytokines play an important role in the expansion of MDSCs under pathological conditions, such as in cancer, trauma, and chronic inflammation (44). In the previous results, we found that the cytokines, IL-6, and IL-10, were significantly increased in the plasma in PMN patients (Figures 5D,E). To understand the relationship among MDSCs, IL-6, and IL-10, and to determine their roles in the occurrence and development of PMN disease, MDSCs were co-cultured with Th2 cells in the presence of 10 ng/mL rIL-6 or rIL-10 in Th2 polarisation conditions (Figure 7C). The results showed that MDSCs from PMN patients, alone or with rIL-6 or rIL-10 stimulation for 24 h, inhibited autologous Th2 polarisation. MDSCs with rIL-6 or rIL-10 stimulation had a stronger ability to inhibit Th2 polarisation compared to untreated MDSCs (Figure 7D). These results imply that IL-6 and IL-10 cytokines enhance the ability of MDSC-mediated Th2 differentiation.

## Discussion

Our study of MDSCs in PMN patients reveals the association of MDSC expansion with disease severity and Th2 and Th17 response. In PMN patients, the MDSCs were significantly increased, Th2 and Th17 immune responses were enhanced, and positively correlated with the disease activity. T cells tended to be more easily polarised into Th2 cells. The MDSCs were significantly more potent in promoting Th17 cell differentiation *in vitro* in an ARG-1-dependent manner and may be related to disease progression of PMN.

MDSCs were significantly increased and positively correlated with plasma anti-PLA2R IgG content and 24-h urine protein quantification in PMN patients. The anti-PLA2R antibody has been recommended as a routine test for all PMN cases, and the titers of anti-PLA2R are correlated with baseline proteinuria, disease activity, and relapse (1, 6, 12, 45, 46). Our data show that 78.26% of PMN patients had positive anti-PLA2R IgG levels, while all healthy volunteers were negative for anti-PLA2R IgG (<20 RU/mL), which further confirmed the high sensitivity and specificity of anti-PLA2R IgG in the diagnosis PMN. Taken together, these data suggest that MDSCs may be a new and valuable marker for diagnosis, monitoring of disease progression, and development of therapeutic strategies for PMN.

Increase in MDSCs promote autoimmune disease progression by contributing to Th17 response (22, 25, 47–51). Recent studies have identified and functionally characterised IL-17-expressing CD4^+^Th17 cells in organ-specific autoimmune diseases (52). The Th17/IL-17 axis significantly contributes to renal tissue damage in murine models of crescentic and proliferative glomerulonephritis (53). IL-17 serum levels correlate with disease activity in patients with ANCA-associated glomerulonephritis or lupus nephritis (54, 55). Moreover, Th17 cells are present in the kidneys of these patients and represent an area of intensive ongoing clinical and basic research (19). However, there are relatively few studies on the relationship between Th17 cells and PMN, and the role of Th17 cells in PMN remains unclear. Our data show that IL-17^+^ cells are adjacent to the CD11b^+^ cells in renal tubules and interstitium in PMN patients as revealed by immunofluorescence. Th17 immune response was enhanced and plasma IL-17A content was significantly increased, and positively correlated with the disease activity in PMN patients. Further, MDSCs were significantly more potent in facilitating Th17 cell differentiation *in vitro* in an ARG-1-dependent manner and may therefore be related to disease progression of PMN. These results suggest that the Th17 response plays a deleterious role in the progression of PMN.

MDSCs express high levels of both enzymes: ARG-1, which converts L-arginine into urea and L-ornithine, and iNOS, which generates NO (20). It had been reported that ARG-1 was involved in immune regulation under physiological and pathological conditions (40, 56, 57). To date, there are very few reports that directly link Arg-1–Th17 responses. Recent data suggest that there is a close correlation between the availability of arginine and the regulation of T-cell proliferation (58). Increased serum Arg-1 activity was found to promote Th17 differentiation both in SLE patients and humanised SLE mice, and was correlated positively with the disease activity. Furthermore, ARG-1–dependent stimulation of Th17 responses by MDSCs is likely mediated by multiple mechanisms involving RORγt, RORα, and mTOR (22). Here, we showed a significant increase in ARG-1 plasma content and a positive correlation with disease activity in PMN patients. MDSCs may promote Th17 cell differentiation *in vitro* in an ARG-1-dependent mechanism, which is consistent with a previous report in SLE patients. However, additional experimental studies including a longitudinal study or an animal model of PMN are needed to better understand the relationship among MDSCs, Th17 cells, and disease progression in PMN.

Cytokines play an important role in the expansion of MDSCs and T-cell differentiation (44). The cytokines secreted by MDSCs influence Th17 polarisation. IL-6 level was elevated in the culture supernatant of MDSCs from the tumour site, and IL-6 neutralising antibody partially inhibited IL-17 production by naïve T cells (59). This suggests that the relative concentration of IL-6 secreted by MDSCs created the ideal environment for Th17 polarisation and IL-17A production. A previous study showed that distinct subsets of myeloid suppressor cells correlate with plasma IL-6 and IL-10, and impaired response to IFN-α to promote anti-tumour immunity in patients with gastrointestinal malignancies (60). Our data show that IL-6 and IL-10, which were elevated in PMN patients, significantly increase ARG-1 production in MDSCs. This is consistent with a previous report which showed that IL-6 induces ARG-1 production in SLE patients (22). First, we demonstrate that the increased plasma levels of IL-6 and IL-10 in the PMN patients further promotes Th17 cell differentiation through the recruitment or expansion of MDSCs and increased production of ARG-1; second, increased MDSCs secrete more IL-6, which in turn promotes Th17 cell differentiation and may be associated with the progression of PMN; third, increased Th2 cytokines (IL-10 and IL-13) may induce ARG-1 synthesis, which also further promote the deterioration of PMN. However, additional experimental confirmation is needed.

Previous studies have shown that Th2 responses are associated with membranous patterns of injury (36). IL-4 production by peripheral Th cells is upregulated and correlated with the severity of proteinuria in PMN patients (15). Our data showed a significant elevation of Th2-related cytokines, such as IL-6, IL-10, and IL-13 in the plasma of PMN patients. However, we found that the level of IL-4 in the plasma were not significantly different between PMN patients and HCs. We propose that an alternate pathway may affect the expression of IL-4, and further experiments are needed to elucidate this mechanism.

Th17 cells represent a pro-inflammatory subset of cells, whereas Tregs have an antagonist effect. Dysregulation of the Th17/Treg balance is critically important for pathogenesis, and is relevant for the prognosis and therapy of autoimmune diseases, including RA, psoriasis, multiple sclerosis (MS), and inflammatory bowel disease (IBD) (61–63). Th17 cells have plasticity and can become Treg and Th2 cells in immune-mediated kidney diseases. Tregs, which play an important role in regulating the balance between Th subsets, are recruited into the inflamed kidneys and protects the organism against overwhelming Th17- and Th1-mediated immune responses in an experimental crescentic glomerulonephritis (nephrotoxic nephritis, NTN) model (64). Data on the peripheral numbers and function of Tregs in human autoimmune diseases are contradictory and remain subject to debate (65–68). In our study, the percentage of Treg cells decreased, and the Th1/Th2 and Th1/Th17 ratios were significantly lower in PMN patients than in HCs (Figure S7). These results suggest that Th1/2/17/Treg immunological imbalance may play an important role in the aetiology and progression of PMN disease.

MDSCs display remarkable plasticity and may inhibit T-cell responses through multiple mechanisms. In humans, MDSCs have no specific markers, and are usually identified through high expression of CD11b or CD33, which are shared by other myeloid cells, such as granulocytes, monocytes, and macrophages. CD11b^+^ cells are used to identify human MDSCs in immunohistochemical and flow cytometry experiment (69). In this study, CD11b was selected as the surface marker for MDSCs. The expansion of MDSCs, as well as their activation and migration, requires different factors in different diseases (70). For example, BM-derived MDSCs are recruited to injured kidneys and protect against acute kidney injury in a mouse model (71). T-cell infiltration is a critical driver of kidney injury (52). Using multiplex IHC staining, our study found that CD3^+^ T cells were adjacent to CD11b^+^ cells in the renal tubules and interstitial areas in PMN patients, which suggests that they are likely to associated with PMN disease.

The expansion of GR-1^+^CD11b^+^ cells *in vivo* contributes to the induction of Th2 polarisation in sepsis (72). MDSCs exacerbated Sjögren's syndrome by inhibiting Th2 cells in NOD mice and SS patients (28). Our data show that Th2 immune responses were enhanced and positively correlated with the disease activity of PMN patients, and that was consistent with the results of previous studies showing that PMN is a Th2-related autoimmune disease (13, 15). However, MDSCs inhibited Th2 cell differentiation through a ARG-1-independent and iNOS-independent mechanism *in vitro*. Compared to untreated cells, MDSCs with rIL-6 or rIL-10 had significantly stronger ability to inhibit autologous Th2 polarisation in PMN patients. This suggests that that MDSCs inhibit autologous Th2 polarisation *in vitro* in association with IL-6 or IL-10 cytokines in PMN patients. Compared to *in vivo*, increased MDSCs and enhanced Th2 response in PMN patients appear to be inconsistent or self-contradicting each other *in vitro*. We speculate that MDSCs may not inhibit Th2 response *in vivo*, which is similar to the role of MDSCs and Th17 response in autoimmune diseases (22, 23, 25). The reasons are as follows. The first reason could be attributed to the plasticity of the phenotype and functions of heterogeneous MDSCs because of the environmental or inflammatory milieu; Secondly, some inflammatory cytokines may also affect the Th2 response *in vivo*, and due to that the inhibition of MDSCs was not enough to completely antagonise the Th2 response and disease progression. Therefore, the positive correlation between MDSCs and Th2 immune responses is not contradictory with the inhibition of Th2 immune responses by MDSCs. In addition, Th2 cytokines, such as IL-4, IL-10, or IL-13, may promote the recruitment of MDSCs and positive correlation between Th2 immune responses and MDSCs in PMN patients. However, the reason for this is unclear and further studies are necessary to elucidate the “functional” interaction between MDSCs and Th2 in different sites.

## Conclusion

In summary, the present study reveals for the first time that MDSCs may be positively correlated with the progression of PMN. MDSCs inhibit the differentiation of Th2 cells *in vitro*. However, MDSCs promote the differentiation of Th17 cells in an ARG-1-dependent, which may be an important factor in the progression of PMN. Our results also suggest that an immunological imbalance in the Th1/Th2/Th17/Treg axis is important in the aetiology and progression of PMN. Further studies are needed to clarify the specific mechanisms involved.

## Data Availability Statement

All datasets generated for this study are included in the article/Supplementary Material.

## Ethics Statement

The studies involving human participants were reviewed and approved by the First Hospital of Jilin University and the Second Hospital of Jilin University. The patients/participants provided their written informed consent to participate in this study. Written informed consent was obtained from the individual(s) for the publication of any potentially identifiable images or data included in this article.

## Author Contributions

HL performed all experiments, analysed the data, and wrote the manuscript. HW, HYu, YX, and ZW were involved in patient recruitment and sample collection. QG and JY were involved in patient recruitment, sample collection, and histological analysis. HYi designed and supervised all experiments and wrote the manuscript. All authors read and approved the final manuscript.

## Conflict of Interest

The authors declare that the research was conducted in the absence of any commercial or financial relationships that could be construed as a potential conflict of interest.
