# Supplementary material for: Myeloid-Derived Suppressor Cells Promote the Progression of Primary Membranous Nephropathy by Enhancing Th17 Response
Source: Front Immunol. 2020 Aug 20;11:1777. doi: 10.3389/fimmu.2020.01777 (PMC7468481; doi:10.3389/fimmu.2020.01777)
Supplement: Supplementary file 1 [file Table_1.docx]

Supplementary Material

**Supplementary Tables**

**Table S1. Demographic and clinical characteristics of the studied groups**

| Number | 29 |
| --- | --- |
| Age (years) | 49.90 ± 17.70 (22–70) |
| Gender (Female/Male) | 9/20 |
| Proteinuria (g/24h) | 10.19 ± 5.46 (2.84–21.28) |
| total protein (g/L) | 45.54 ± 7.75 (33.60–57.60) |
| Serum album (g/L) | 21.08 ± 5.59 (10.7–31.3) |
| Urea nitrogen (mmol/L) | 4.75 ± 2.59 (1.84–13.82) |
| Serum creatinine (μmol/L) | 72.85 ± 24.23 (35.3–154.00) |
| α1 microglobin (mg/L) | 44.85 ± 13.21 (21.78–71.42) |
| β2 microglobin (mg/L) | 2.03 ± 1.01 (0.22–5.23) |
| Clinical stage | I-II phase |
| IgG4 | 2+ – 4+ |

**TableS2. Patients’ characteristics at diagnosis (n = 29)**

| Patient | Gender | Age | Proteinuria (g/24h) | IgG4 | C3 | Pathological staging |
| --- | --- | --- | --- | --- | --- | --- |
| 1 | M | 64 | 6.37 | 3+ | 2+ | I-II |
| 2 | M | 22 | 10.42 | 2+ | - | II |
| 3 | M | 55 | 7.72 | 2+ | - | I-II |
| 4 | M | 37 | 11.83 | 2+ | - | I-II |
| 5 | F | 45 | 2.84 | 2+ | - | I-II |
| 6 | M | 64 | 4.77 | 2+ | - | II |
| 7 | M | 26 | 17.15 | 4+ | + | II |
| 8 | F | 49 | 3.06 | 3+ | + | II |
| 9 | M | 56 | 11.79 | + | + | I-II |
| 10 | F | 61 | 13.92 | 4+ | 3+ | I-II |
| 11 | F | 70 | 5.36 | 2+ | - | I |
| 12 | M | 60 | 10.92 | 2+ | - | II |
| 13 | F | 56 | 3.43 | 2+ | 2+ | I |
| 14 | M | 65 | 3.95 | 2+ | - | I-II |
| 15 | M | 26 | 11.1 | 2+ | 2+ | II |
| 16 | M | 60 | 14.41 | 2+ | - | I-II |
| 17 | F | 47 | 7.82 | 2+ | + | I |
| 18 | M | 24 | 17.77 | 2+ | 2+ | I-II |
| 19 | M | 56 | 14.97 | 2+ | 2+ | I |
| 20 | M | 56 | 18.56 | 2+ | + | II |
| 21 | M | 62 | 10.03 | 2+ | - | I |
| 22 | F | 27 | 14 | 2+ | - | II |
| 23 | M | 51 | 12.02 | 2+ | - | II |
| 24 | M | 52 | 8.24 | 2+ | 2+ | I-II |
| 25 | F | 53 | 2.99 | 2+ | - | I |
| 26 | M | 52 | 21.28 | 2+ | + | I |
| 27 | M | 39 | 4.6 | 2+ | + | I-II |
| 28 | F | 51 | 6.7 | 2+ | + | I-II |
| 29 | M | 61 | 19.44 | 2+ | - | I |

C3: Component 3; IgG4: Immunoglobulin G4

| **Table S3. Antibodies used for multiplexed immunohistochemistry staining** | | | | | |
| --- | --- | --- | --- | --- | --- |
| **Order** | **Antibody** | **Host (clone); company** | **Dilution** | **Incubation/temp** | **TSA dyes** |
| 1 | CD3 | Rabbit; Abcam | 1:100 | Overnight/4℃ | 570 |
| 2 | CD11b | Rabbit (EPR1344); Abcam | 1:100 | 60 min/RT | 520 |
| 3 | IL-4 | Rabbit; Abcam | 1:100 | 60 min/RT | 650 |
| 4 | CD66b | Rabbit; Abcam | 1:200 | 60 min/RT | 620 |
| 5 | CD14 | Rabbit; Abcam | 1:200 | 60 min/RT | 690 |
| 6 | DAPI | Perkin Elmer Opal 7-color kit | 2 drops/ml | 5 min/RT |  |

TSA dyes: Tyramide Signal Amplification dyes; DAPI: 4’,6-diamidino-2-phenylindole
