# Supplementary material for: Myeloid-Derived Suppressor Cells Promote the Progression of Primary Membranous Nephropathy by Enhancing Th17 Response
Source: Front Immunol. 2020 Aug 20;11:1777. doi: 10.3389/fimmu.2020.01777 (PMC7468481; doi:10.3389/fimmu.2020.01777)
Supplement: Supplementary file 2 [file Data_Sheet_1.docx]

Supplementary Material

## Supplementary Figures


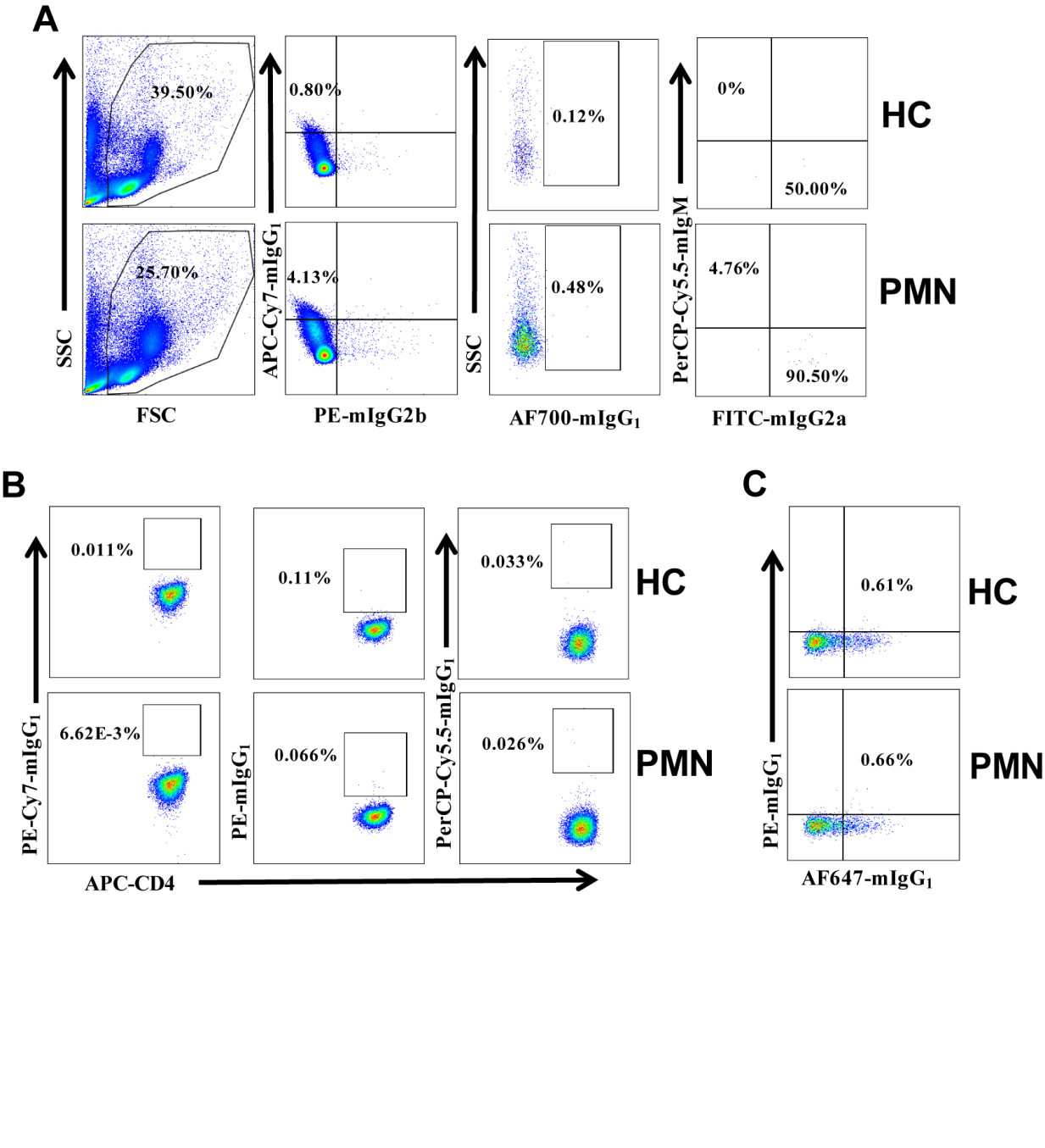


**Figure S1. Isotype controls and gating strategy for flow cytometry analysis**

**(A)** Isotype control and gating strategy for analyzing MDSCs. **(B)** Isotype control for analyzing Th1/2/17 cells in freshly isolated PBMCs. **(C)** Isotype control for analyzing Treg cells in freshly isolated PBMCs.


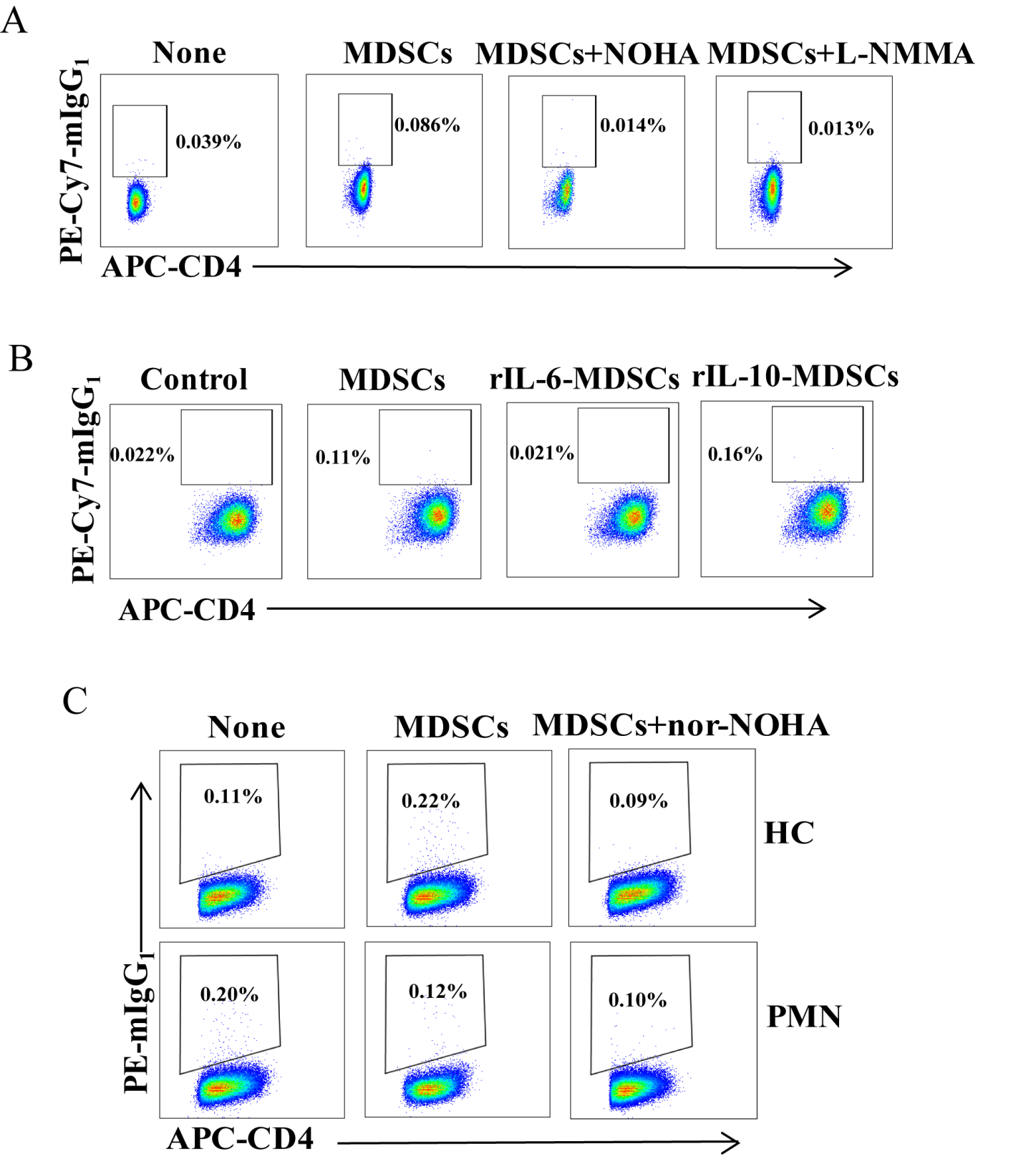


**Figure S2. Isotype controls and gating strategy for flow cytometry analysis**

**(A and B)** Isotype controls for intracellular staining of IL- 4 in polarized CD4^+^ Th2 cells. **(C)** Isotype controls for intracellular staining of IL-17A in polarized CD4^+^ Th17 cells.


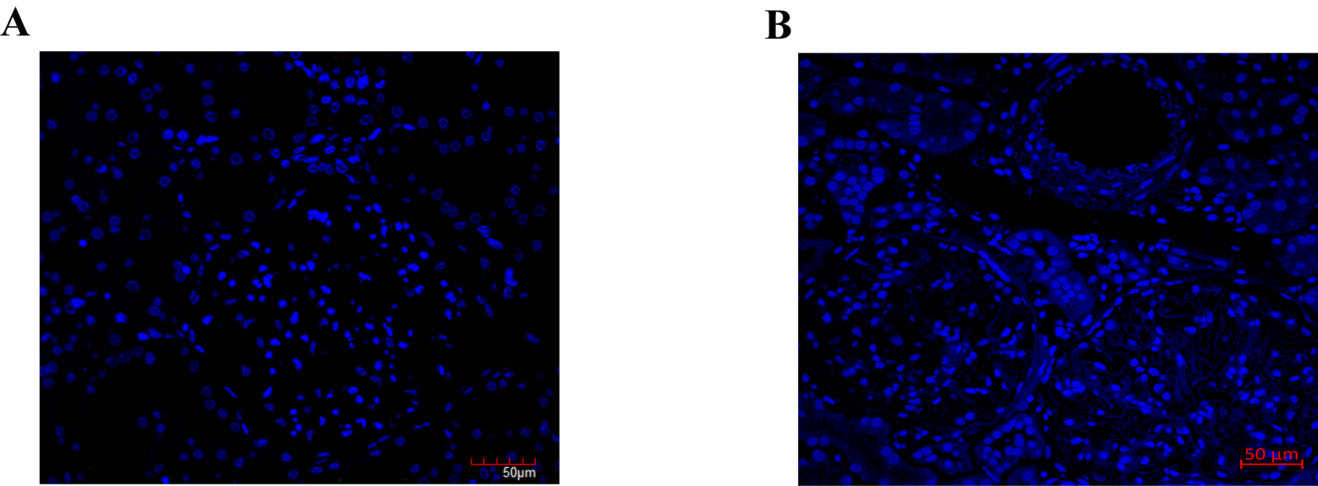


**Fig. S3. Isotype and negative controls for immunofluorescence and multiplexed IHC staining. (A)**Isotype control staining for IL-17(rabbit IgG) and CD11b (rat IgG2b)in human renal tissue sections. Scale bars represent 50μm. **(B)** Negative controls with secondary antibody only but without addition of primary antibody for multiplex IHC. Scale bars represent 50μm.


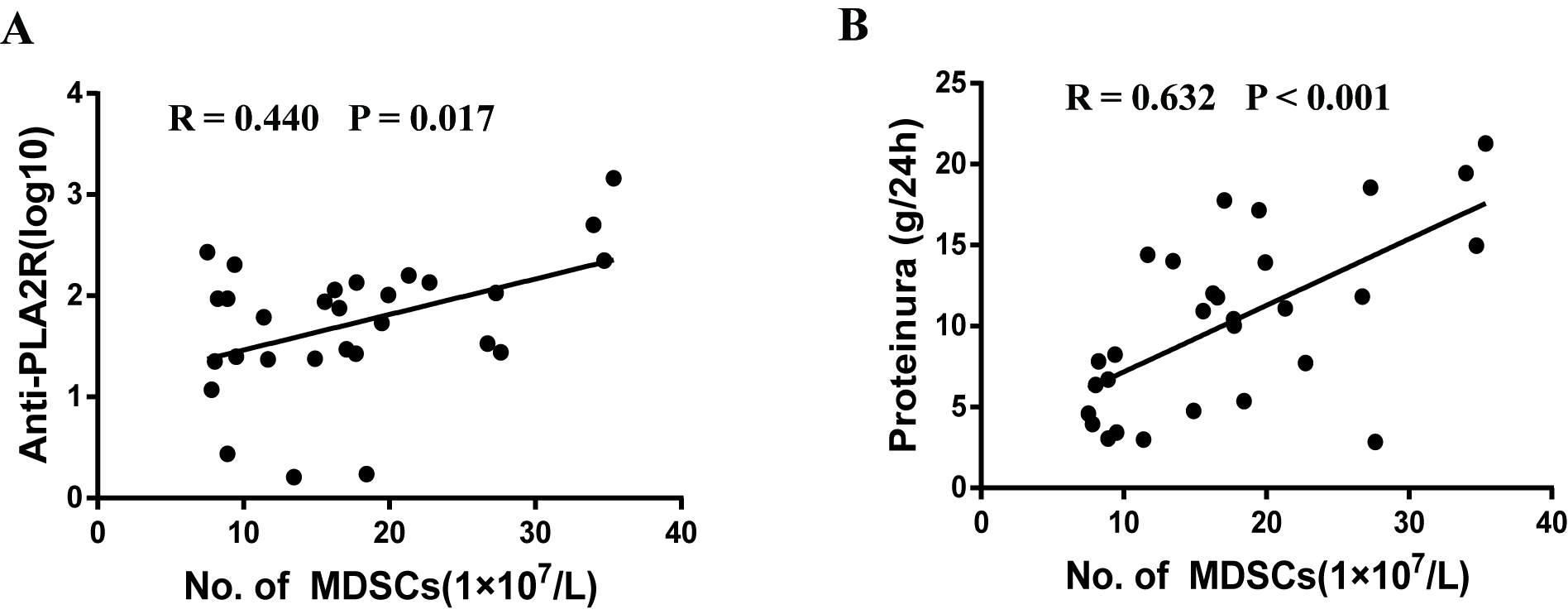


**Fig. S4. Correlation between MDSCs numbers and the disease activity of PMN patients.**

**(A and B)** The correlation analysis between MDSCs numbers and Anti-PLA2R levels (P = 0.017, Pearson correlation) **(A)**  and 24-hour urine protein quantification (P < 0.001, Pearson correlation) **(B)** from PMN patients. Anti-PLA2R is displayed with log10.


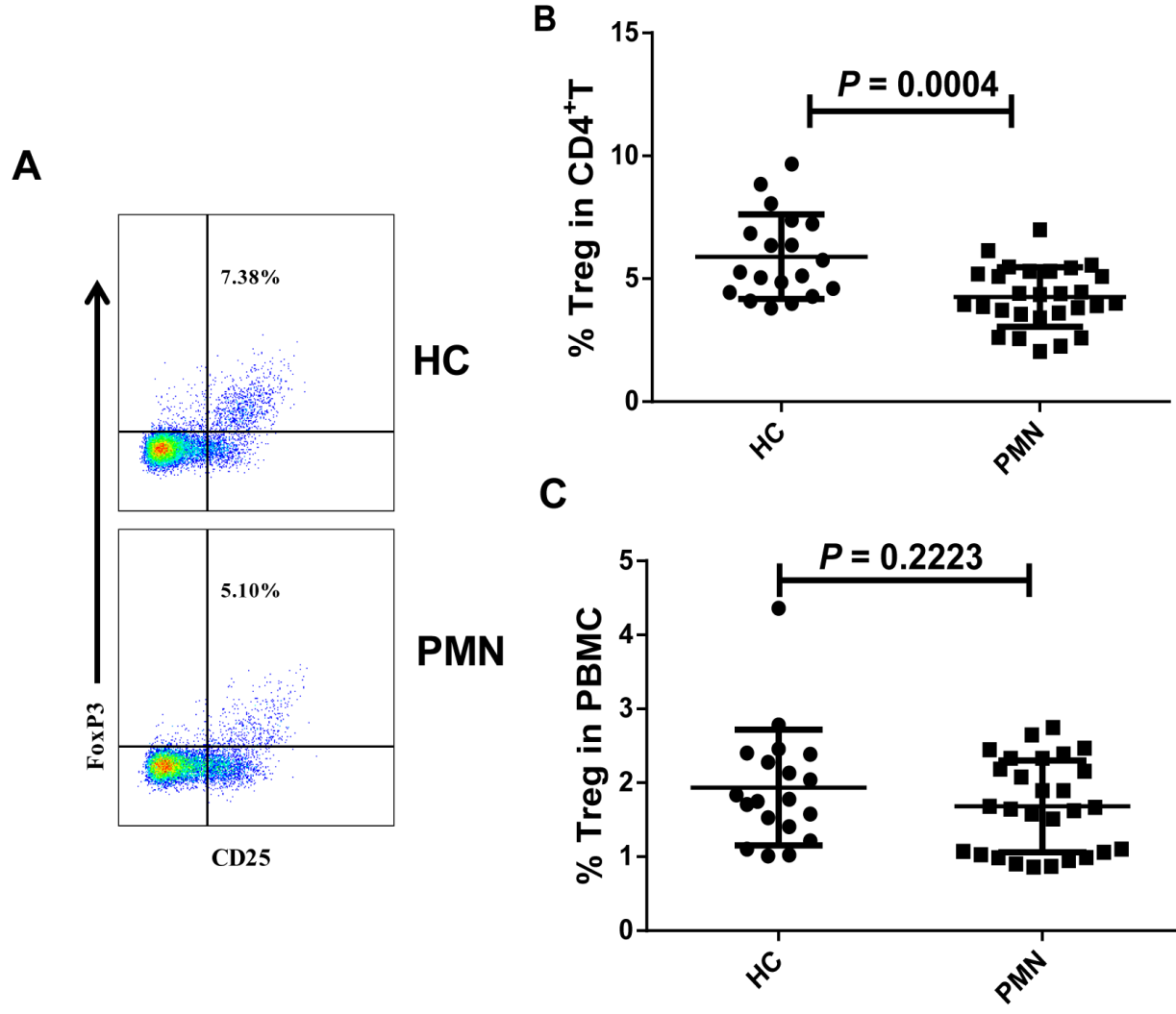


**Figure S5.** **Treg cells from HCs and PMN patients**

**(A)** Staining profiles of Tregs (CD4^+^CD25^+^Foxp3^+^) from the peripheral blood of a representative HC and a representative PMN patient. **(B)** The percentages of Tregs in CD4^+^ T (*P* = 0.0004, 5.896 ± 1.721% versus 4.255 ± 1.217%, *t* test; upper panel) or PBMCs (*P* = 0.2223, 1.935 ± 0.784% versus 1.681 ± 0.620%, *t* test; lower panel) from the peripheral blood of HCs and PMN patients.


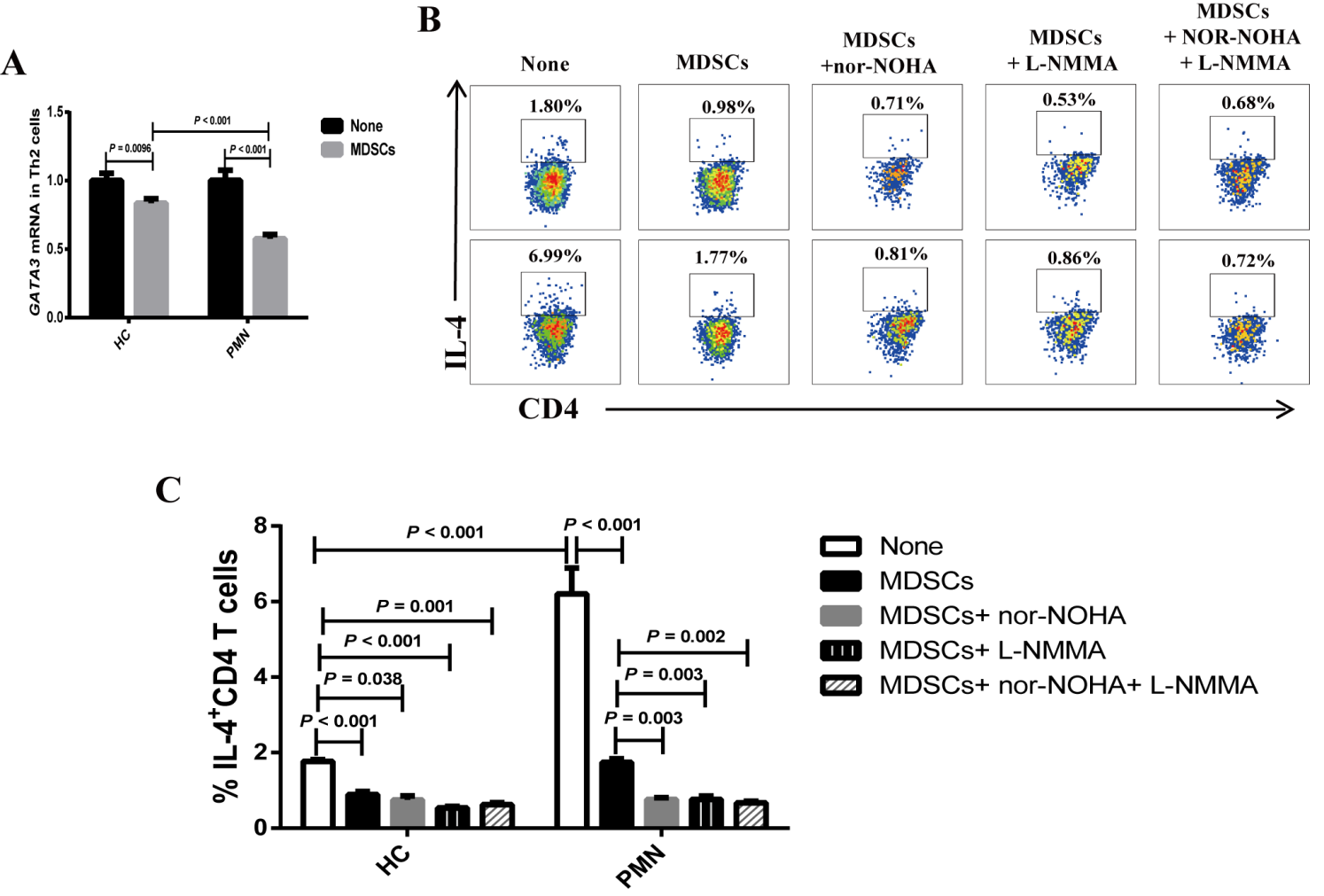


**Fig S6. The inhibitory ability of MDSCs-mediated Th2 differentiation are ARG-1-independent and iNOS- independent.**

**(A)**Naïve CD4^+^ T cells from HCs and PMN patients were cultured under Th2 differentiation without

MDSCs and with autologous MDSCs, and analyzed the expression of *GATA3* mRNA, i.e., specific transcription factor of the Th2 cells in HCs (*P* = 0.0096, *t* test) and PMN patients (*P* < 0.001, *t* test), and HC-MDSCs versus PMN-MDSCs (*P* < 0.001, *t* test) by qRT-PCR. **(B and C)** Naïve CD4^+^ T cells from HCs and PMN patients were cultured under Th2 differentiation without MDSCs, with autologous MDSCs, with autologous MDSCs + nor-NOHA or with autologous MDSCs + L-NMMA, or with both and analyzed the expression of IL-4^+^CD4^+^ T cell. Shown are representative staining profiles**(B)** and percentages **(C)** of IL-4^+^CD4^+^ T cell from HCs (Th2 versus MDSCs, *P* < 0.001; MDSCs versus MDSCs + nor-NOHA, *P* = 0.038; MDSCs versus MDSCs + L-NMMA, *P* < 0.001; MDSCs versus MDSCs + both, *P* = 0.001, ANVOA) and PMN patients (n = 3; Th2 versus MDSCs, *P* < 0.001; MDSCs versus MDSCs + nor-NOHA, *P* = 0.003; MDSCs versus MDSCs + L-NMMA, *P* = 0.003; MDSCs versus MDSCs +both, *P* = 0.002, ANVOA), and HC-Th2 versus PMN-Th2, *P* < 0.001, ANVOA.

**
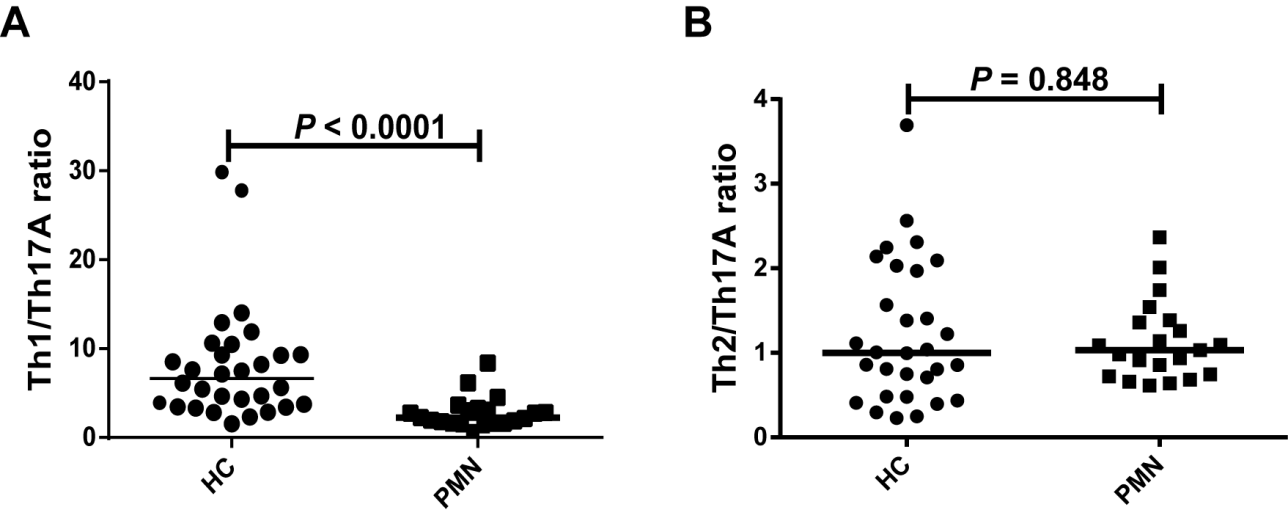
**

**Figure S7. Th1/Th17 and Th2/Th17 ratios from PBMCs of HCs and PMN patients**

**(A)** The Th1/Th17 ratio (*P* < 0.0001, Mann-Whitney U test; 4.698 (3.428, 8.766) versus 2.246 (1.553, 3.182)) and **(B)** Th2/Th17 ratio (*P* = 0.848, Mann-Whitney U test; 0.810 (0.422, 1.301) versus 1.0308 (0.733, 1.372)) in the HCs and PMN.
